# Ciprofloxacin (Zwitterion, Chloride, and Sodium Forms): Experimental and DFT Characterization by Vibrational and Solid-State NMR Spectroscopies

**DOI:** 10.1021/acsomega.5c06384

**Published:** 2025-10-11

**Authors:** Filipe C. D. A. Lima, Arthur P. Camargo, Fabrice Leroux, Jocelyne M. Brendlé, Marcia L. A. Temperini, Helena M. Petrilli, Vera R.L. Constantino

**Affiliations:** † Instituto Federal de Educação, Ciência E Tecnologia de São Paulo (IFSP), Campus Matão, Av. Stefano D’avassi, 625, Matão, SP CEP 15991-502, Brazil; ‡ Departamento de Física dos Materiais e Mecânica, Instituto de Física, 28133 Universidade de São Paulo (USP), Rua Do Matão, 1371, São Paulo, SP CEP 05508-090, Brazil; § 27006 Institut de Chimie de Clermont-Ferrand ICCF, CNRS UMR 6296, Université Clermont Auvergne, 24 Avenue Blaise Pascal, Clermont-Ferrand F-63000, France; ∥ Institut de Science des Matériaux de Mulhouse, CNRS UMR 7361, Université de Haute-Alsace, 15 Rue Jean Starcky, Mulhouse F-68100, France; ⊥ Université de Strasbourg, 20A Rue René Descartes, Strasbourg F-67081, France; # Departamento de Química Fundamental, Instituto de Química, Universidade de São Paulo (USP), Av. Prof. Lineu Prestes 748, São Paulo, SP CEP 05508-000, Brazil

## Abstract

Ciprofloxacin (Cipro), a widely used fluoroquinolone antibiotic, exists in multiple protonation states, which influence its structural and spectroscopic properties. Despite its pharmaceutical relevance and concerns regarding its accumulation in the environment, a comprehensive characterization of its zwitterionic, cationic, and anionic solid forms remains limited, particularly in terms of their vibrational and nuclear magnetic resonance (NMR) spectral assignments. The focus of this study was to identify spectral signatures that differentiate Cipro and its cationic form (as a chloride salt) and anionic form (as a sodium salt). All samples were characterized in solid-state using X-ray diffraction, thermogravimetric analysis coupled with mass spectrometry, infrared and Raman spectroscopies, and cross-polarized magic-angle spinning (CP-MAS) solid-state NMR, with support from density functional theory (DFT) calculations. Both salts were synthesized in this study. The cipro sodium was isolated as a monohydrate salt, a previously unreported phase. Six spectral regions were identified to distinguish the Cipro zwitterion from its cationic and anionic forms by using vibrational spectroscopy. Both experimental ^13^C CP-MAS solid-state NMR and theoretical analyses revealed pronounced chemical shifts induced by protonation and counterion interactions, which also differentiate the three forms. The analysis presented here provides clear fingerprints of the three Cipro forms, which can be used to support reference spectroscopic data, with direct implications in pharmaceutical formulations as well as for environmental studies.

## Graphical abstract

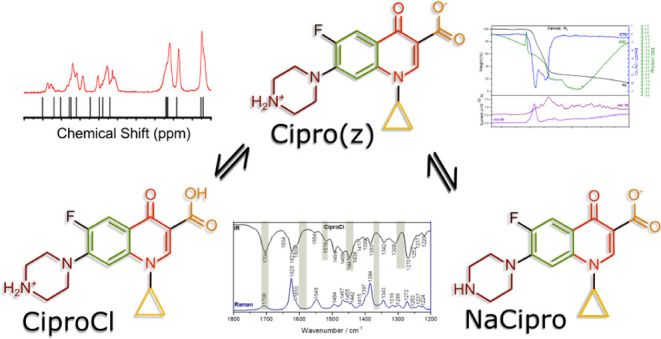

## Introduction

Ciprofloxacin (Cipro) is a synthetic, second-generation quinolone antibiotic with broad-spectrum activity, introduced to the clinic in 1987, whose mechanism of action involves the inhibition of DNA synthesis.^1^ According to the World Health Organization (WHO), ciprofloxacin is an essential medicine for the primary healthcare system that should be widely available, accessible, and of high quality.^2^ However, the WHO emphasizes that quinolone and fluoroquinolone antibiotics should be administered in the most critical diseases due to the potential risk of developing drug-resistant bacteria. Cipro (1-cyclopropyl-6-fluoro-4-oxo-7-(piperazine-1-yl)-quinoline-3-carboxylic acid) is a fluoroquinolone (Figure 1a) having a 6-membered nitrogen heterocycle (piperazine group in a chair conformation) and a cyclopropyl group.^3^ Crystals comprising non-ionized or neutral molecules of Cipro interacting by hydrogen bonds could be obtained through several cycles of vaporization and condensation steps.^2^ However, the non-ionized Cipro solid is very hygroscopic. It is transformed into its zwitterion form, Cipro (z), through a proton transfer from the carboxyl group to the amine of the piperazine group (Figure 1b).^3^ Heating Cipro­(z) before its melting point (around 285 °C) produces neutral Cipro molecules.^4^ Considering the characteristics of neutral Cipro above, the regularly available product is in the zwitterion form. The amorphous Cipro can be obtained by spray drying from water.^4^

**Figure 1:**
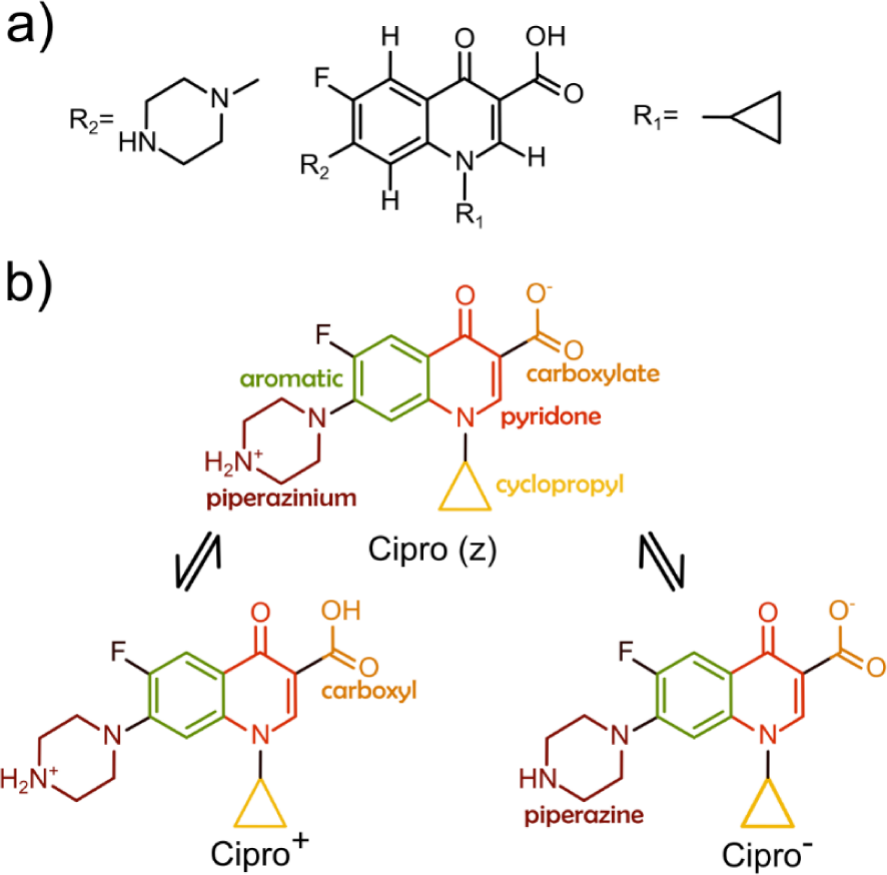
(a) General fluoroquinolone structure; R_1_ = cyclopropyl group and R_2_ = 6-membered nitrogen heterocycle (piperazine group); (b) ciprofloxacin in the zwitterion form (Cipro­(z)), cation form (Cipro^+^), and anion form (Cipro^–^).

Cipro­(z) molecules are identified in the anhydrate crystal, forming centrosymmetric dimers in which the positive piperazinium group interacts with the negative carboxylate group of adjacent species.^3,5^ The anhydrous solid can be hydrated when suspended in water or exposed to high humidity conditions.^4^ Cipro­(z) monocrystals can be obtained as hexahydrate^6^ or 3.7 hydrate^7^ forms, or as a methanol hemisolvate.^8^

Three Cipro forms can be generated in aqueous medium, as shown in Figure S1. The solubility of ciprofloxacin is dependent on the pH value of the solution. Cipro acid dissociation constants (expressed as pK_a_) of a carboxyl group and the nitrogen of the piperazinium group are 6 and 8.6, respectively.^7,9^ At pH values lower than about 5, the piperazine basic atom and carboxylate group are protonated, producing cationic species (Cipro^+^; Figure 1b); the zwitterion is the main species in the pH range of approximately 5–8.5; at pH values higher than 9, piperazinium group is deprotonated giving anionic species (Cipro^–^; Figure 1b) in solution. Typically, Cipro is commercialized in the form of hydrochloride monohydrate (CiproCl·H_2_O), whose solubility is higher than that of its anhydrous zwitterionic form (30.0 mg mL^–1^ versus 0.06 mg mL^–1^ at 25 °C, respectively).^7,10^ Several strategies have been investigated to improve the solubility, bioavailability, and permeability of Cipro in the organism.^11^

Salts of cationic ciprofloxacin (Cipro^+^) can be obtained by having the counterion chloride,^12^ dihydrogenphosphate,^13^ organic anions such as lactate^14^ or fumarate,^15^ or metal complexes such as [ZnCl_4_].^2–16^ On the other hand, sodium salts of anionic ciprofloxacin (Cipro^–^) pentahydrate or hexahydrate were crystallized using high-pressure crystallization.^5^

Considering the pharmacological activity of Cipro, the carboxylate and ketone groups of quinolones are responsible for interacting with the DNA bases of bacteria.^17^ The interest in identifying Cipro forms, interfacial interactions, and potential metal coordination in different environments goes beyond the biological sphere. For example, Cipro can be confined in structures known as drug carriers,^18–23^ associated with metal nanoparticles for improved action,^1^ undergoes sorption into/on minerals in the soil after discharge,^21,24–26^ or be eliminated using adsorbent materials for the removal of emerging contaminants from water.^27–30^

Spectroscopic methods, such as vibrational spectroscopy, electronic ultraviolet and visible (UV-vis) spectroscopy, or nuclear magnetic resonance (NMR), are suitable tools to investigate the interactions among Cipro forms and other species (lipids, minerals, biomolecules, etc.) to obtain information about the groups involved in the interfaces. However, the vibrational spectra of quinolones exhibit several bands that challenge the unequivocal assignment of vibrational modes. Modifications in the IR (vibrational) and UV-vis (electronic) spectral profiles of Cipro as a function of pH value, from a basic solution to an acidic solution, were reported.^27^ The ^13^C-NMR data were reported only for the zwitterionic Cipro form.^4,31^ Although some computational calculations have been done to attribute the Cipro vibrational bands, the focus was not on identifying the Cipro forms through characteristic band assignments. Density functional theory (DFT) calculations using different functionals and bases were employed to obtain the infrared (IR) and Raman spectra of Cipro in its neutral state,^32^ the UV-vis spectra and atomic orbitals,^33^ and the possible protonated forms of Cipro.^34^ The IR and Raman spectra were simulated for Cipro’s cationic^33^ and zwitterionic^12,34,35^ forms.

In the present study, the spectroscopic properties of commercial Cipro, its cationic form as a chloride salt (abbreviated CiproCl), and its anionic form as a sodium salt (abbreviated NaCipro) were compared to identify the protonation level of Cipro. Both salts were prepared and isolated in a solid state. Before the spectroscopic investigation, the three solid Cipro forms were characterized by X-ray diffractometry (XRD) and thermogravimetric analysis coupled to mass spectrometry (TGA-MS) in both air and nitrogen atmospheres. The experimental characterization of Cipro is crucial because it can exhibit polymorphism and may exist in crystalline form as zwitterions or non-ionized species. Additionally, it can form solvates and hydrates. If the identities of these chemical forms are not well established, it could compromise the integrity of simulated spectroscopic studies.

The theoretical spectra of the three Cipro forms were simulated in a vacuum to assign their experimental vibrational and ^13^C NMR spectra. Although some studies have been conducted on the vibrational and NMR spectroscopic profiles of Cipro forms (primarily in solutions), the focus has not been on comparing them to search for spectral signatures that differentiate their protonation levels. Considering the support of the calculations for assigning the experimental vibrational spectrum, most reported studies involved the non-ionized (or neutral) form of ciprofloxacin, the less available form of this drug. To the best of our knowledge, the vibrational experimental and calculated spectra of an anionic salt of Cipro in the solid state are reported for the first time in this work.

## Experimental and Computational Details

### Preparation of Cipro Forms

Cipro anhydrous (C_17_H_18_FN_3_O_3_, 98%) and sodium hydroxide (NaOH, 98%) from Aldrich, and hydrochloric acid (HCl, 36.5–38%) from Synth, were used as received. Cipro was prepared in both chloride and sodium salt forms by stoichiometric neutralization with standard aqueous HCl and NaOH solutions, respectively, and isolated by lyophilization using the Thermo Savant ModulyoD equipment.

### Characterization of Cipro Samples

XRD patterns of powdered samples were recorded on a Rigaku diffractometer, model Miniflex, using CuK_α_ radiation (1.541 Å, 30 kV, 15 mA, scan step of 0.03^o^) and a Ni filter. Mass-coupled thermal Analyses (Thermogravimetric Analysis and Mass Spectrometry, TGA-MS) were recorded on a Netzsch thermoanalyser model TGA/DSC (Differential Scanning Calorimetry) 490 PC Luxx, coupled to an Aëolos 403 C mass spectrometer. The analyses were conducted using an aluminum crucible under a synthetic air or nitrogen flow of 50 mL/min and a heating rate of 10 °C/min. Fourier transform Raman (FT-Raman) spectra were recorded in an FT-Raman Bruker FRS-100/S spectrometer using 1064 nm exciting radiation (Nd:YAG laser Coherent Compass 1064–500 N), laser power of 100 mW, Ge detector, in the spectral range of 3500–50 cm^–1^, resolution of 4 cm^–1^, and 256 scans. Infrared vibrational spectra were recorded using the attenuated total reflectance mode on a Bruker Alpha spectrometer, in the spectral range of 4000–400 cm^–1^, with a resolution of 4 cm^–1^ and 128 scans. ^13^C solid-state NMR spectra were recorded on a 300 Bruker Advance spectrometer operating at 75.47 MHz. A cross-polarization (CP) sequence, transferring magnetization of ^1^H nuclei toward ^13^C nuclei, was used under magic angle spinning (MAS) conditions operating at 10 kHz. The Hartman-Han contact time to allow the transfer was 1 ms, making the intensity of the ^13^C chemical shifts highly dependent on their spatial environment in protons, i.e., leading to a non-quantitative nuclei response under CP conditions. Spinel 64 ^1^H phase-decoupling was applied during acquisition, and a recycling time of 5 s was used. The spectra are referenced to the carbonyl of glycine, calibrated at 176.03 ppm with 2.000 scans to get a proper signal-to-noise ratio.

### Density Functional Theory Calculations

The initial atomic positions of each simulation were obtained from monocrystal X-ray diffraction data as follows: CiproCl structure^12^ for hydrated ciprofloxacin hydrochloride (Cambridge Crystallography Data Base, CCDC 213552); Cipro­(z) and NaCipro structures^5^ for anhydrous ciprofloxacin (*CCDC* 714344) and ciprofloxacin sodium pentahydrate (*Crystallography Open Database, COD* 7200856). All simulations were performed in the Kohn-Sham scheme for the DFT,^36^ as implemented in Gaussian 09.^37^ The B3LYP exchange-correlation functional^38,39^ was employed in combination with the basis set 6-311G­(d,p).^40^ In the CiproCl structure, a chloride ion was positioned close to the piperazinium group. A sodium counter was positioned between the carboxylate and carbonyl groups in the NaCipro structure (see Figure 1b). The geometry optimization was performed using a self-consistent cycle to reach a local minimum in the potential energy surface. The harmonic approximation was used to calculate vibrational modes, and visually inspected computer-animated molecular motions were performed to support the vibrational assignments. The computed vibrational wavenumbers were scaled by a factor of 0.985. In addition, the ^13^C NMR chemical shift calculations were performed using the Gauge-Including Atomic Orbital (GIAO) method^15,41,42^ and the diffuse 6–311++G­(d,p) basis set.

## Results and Discussion

### Sample Characterization by XRD and Thermal Analysis

The XRD patterns of Cipro­(z) commercial and the CiproCl and NaCipro samples prepared in this work are shown in Figure S2. All samples are crystalline, but the XRD pattern of NaCipro showed a halo at about 25–30 ° scattering angles, indicating a partially amorphous phase. The XRD patterns of Cipro were compared to the simulated XRD data obtained from the literature,^5,6^ using the Visualization for Electronic and Structural Analysis (VESTA) program (Figure S3), endorsing that the Cipro­(z) sample is anhydrous and contains zwitterion species. The CiproCl sample isolated in this work exhibited an XRD profile (Figure S4) that was very similar to that reported for ciprofloxacin hydrochloride 1.34-hydrate.^12^ Table S1 indicates the experimental interplane distance values for Cipro­(z) and CiproCl samples and the values from monocrystal structures for comparison purposes. The XRD pattern of NaCipro differed from that reported for sodium salts (Figure S5), which were obtained at high pressure,^5^ indicating the isolation of a different arrangement when prepared under ambient conditions. XRD data for NaCipro are compiled in Table S1.

Figure 2 displays the TGA/DTG, DSC, and MS curves of Cipro­(z) under synthetic air and nitrogen atmospheres. The sample could be considered anhydrous, as the mass loss was approximately 1.8% below 100 °C under air and zero in the nitrogen atmosphere, indicating the presence of water from ambient moisture (no water of crystallization). The endothermic peak at 270 °C could be assigned to the melting point of Cipro­(z). According to the literature,^15^ anhydrous zwitterionic ciprofloxacin melts around 270.7 °C. The melting process was followed by the decomposition of Cipro­(z) in both atmospheres, producing H_2_O (*m/z* 18) and CO_2_ (*m/z* 44), as indicated by the MS curves (Figure 2). Under the experimental conditions used in this work, fragments related to HF or nitrogen oxides were not detected. Above 500 °C, the products of Cipro­(z) decomposition in the 300- 500 °C range were decomposed under air conditions.

**Figure 2:**
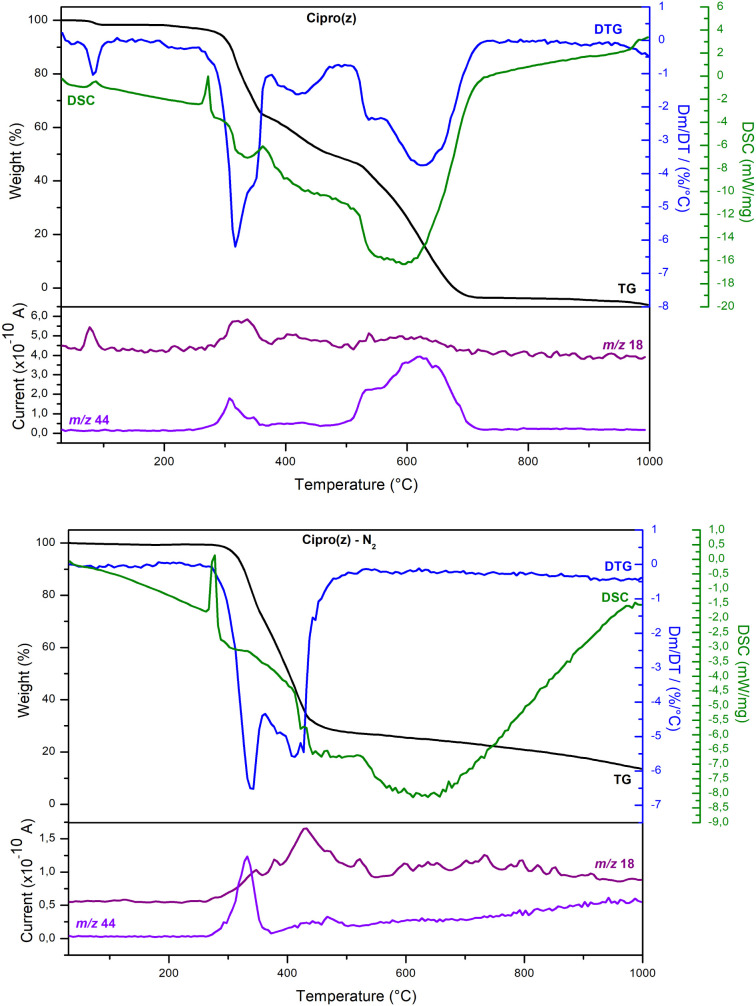
TGA (black), DTG (blue), DSC (green), and MS curves for Cipro­(z) from Aldrich under a synthetic air atmosphere (top) and a nitrogen atmosphere (bottom).

Figure 3 shows the TGA/DTG, DSC, and MS curves of CiproCl under synthetic air and nitrogen atmospheres. Between 100–140 °C in both atmospheres, the sample lost approximately 5.6% of H_2_O (*m/z 18*) (endothermic event), corresponding to the (C_17_H_19_FN_3_O_3_)­Cl·1.2H_2_O composition. The obtained formula was close to that reported in the literature, (C_17_H_19_FN_3_O_3_)­Cl·1.34H_2_O.^12^ In the 280°C-400 °C range, CiproCl lost H_2_O (*m/z* 18) and CO_2_ (*m/z* 44) in an endothermic event at about 320 °C, followed by an exothermic one at about 350–400 °C. Additionally, the sample released HCl (*m/z* 36) at around 300 °C under an air atmosphere and underwent a second prominent decomposition step at approximately 450°C-600 °C. Above 450 °C under nitrogen atmosphere, the mass loss was low up to 1000 °C, producing approximately 20% of residue. The fragments from HF and nitrogen oxides were not observed in either atmosphere under the experimental conditions employed in this work.

**Figure 3:**
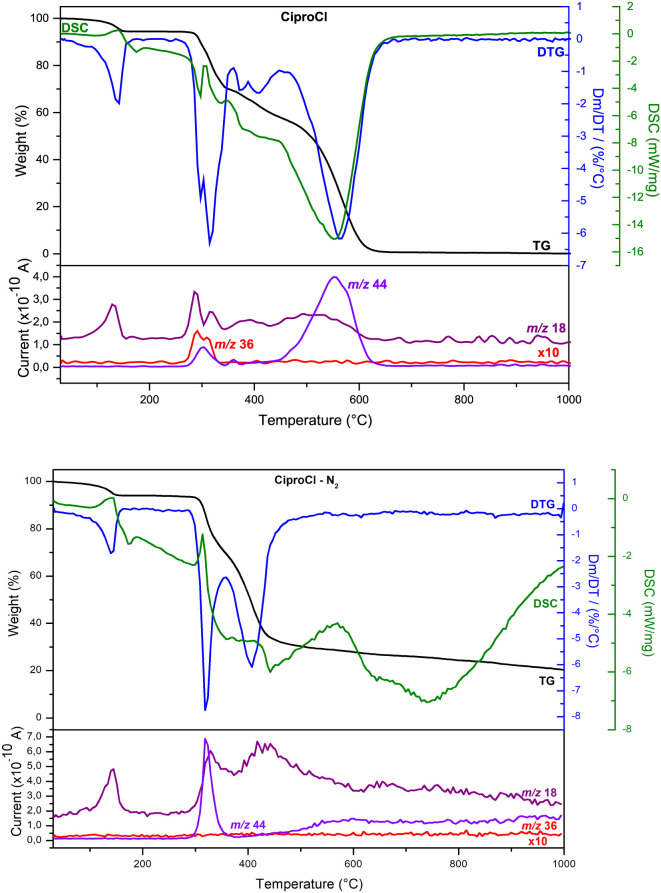
TGA (black), DTG (blue), DSC (green), and MS curves of CiproCl under synthetic air atmosphere (top) and nitrogen atmosphere (bottom).

Figure 4 shows the thermal analysis data of NaCipro under synthetic air and nitrogen atmospheres. The DSC curve did not show an event that could be assigned to melting or phase transition. All DSC signals were related to mass loss steps. The water released up to 150 °C in both atmospheres was associated with the Na­(C_17_H_17_FN_3_O_3_)·H_2_O (371 g mol^–1^) composition, a previously unreported monohydrate salt. These results are important because hydrates can have different physical and chemical properties compared to the other forms of the drug, potentially affecting its stability, solubility, and bioavailability. Considering the mass loss in the air atmosphere and the nature of the released fragments, a thermal decomposition proposal is presented in Scheme S1. The NaCipro sample is decomposed to the NaF compound. There were no reported studies to compare the results presented here.

**Figure 4:**
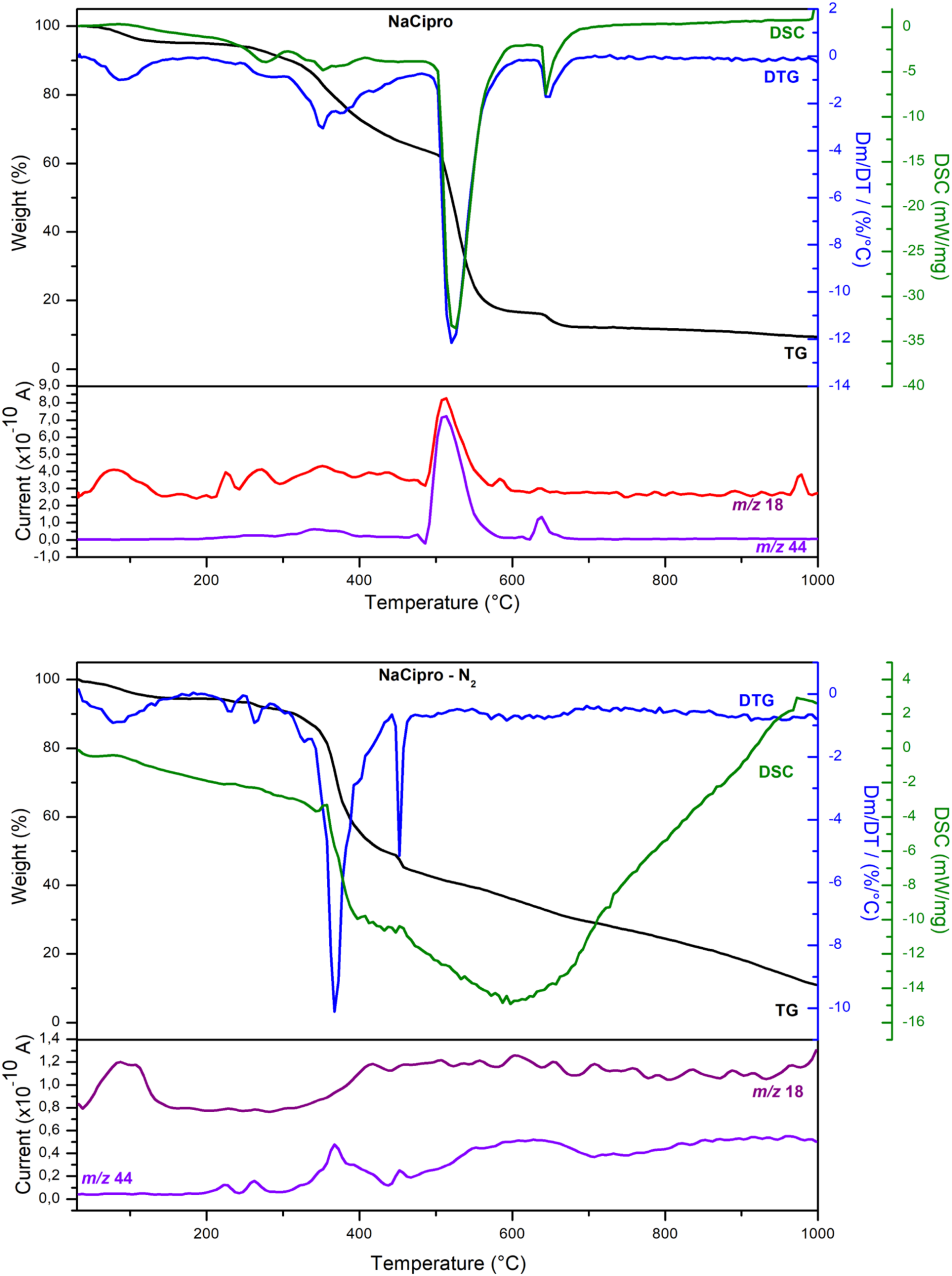
TGA (black), DTG (blue), DSC (green), and MS curves of NaCipro under synthetic air atmosphere (top) and nitrogen atmosphere (bottom).

### Structural Simulations of Cipro Forms by DFT

Figure 5 illustrates the overlapping images of the structures determined from the monocrystal data and the obtained optimized geometries for CiproCl, Cipro­(z), and NaCipro. The overlap indicates that the DFT results accurately reproduce crystallographic geometries. The obtained theoretical structure of NaCipro indicated the coordination of sodium to one oxygen from the carboxylate group and the oxygen of pyridone, as observed in the monocrystal structure.^5^

**Figure 5:**
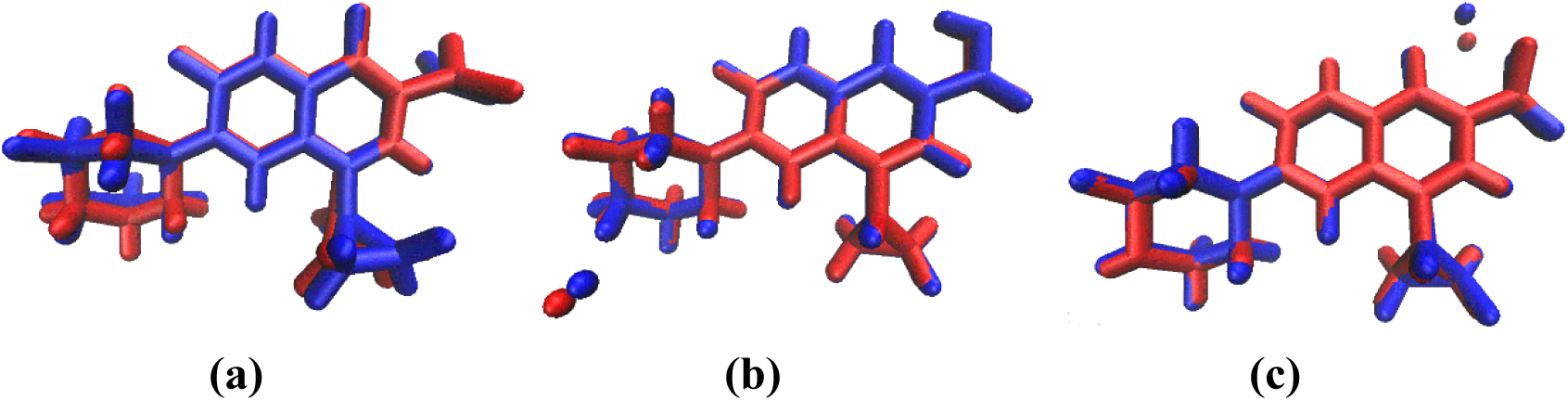
Overlap of DFT-optimized structures (blueao5c06384_0005.tif line) of (a) CiproCl, (b) Cipro­(z), and (c) NaCipro, and those obtained by X-ray structural resolution (red line).

The numerical data, including bond lengths and torsion angles, are presented in Table 1; the bond lengths agree with experimental values, with an average deviation of 0.01–0.05 Å. The most significant differences were observed in the carboxylate region, particularly in the C12–O bonds, where electronic effects related to protonation and counterion interactions influence bond order and length. Similarly, variations in the C3–C12 bond reflect the conjugation changes associated with the deprotonation of the carboxyl group. Torsion angles further illustrate the impact of protonation and counterion on the molecular conformation. The dihedral angle τ_1_ (C6C7N2C15), associated with the piperazine ring, remained nearly identical in CiproCl and NaCipro but undergoes a significant change in Cipro­(z), increasing from 161.7° to 177.5°. This variation suggests that the zwitterionic form stabilizes an extended conformation, reducing steric interactions. Conversely, the τ_2_ (C2N1C9C11) angle, which defines the orientation of the cyclopropyl group, remains consistent in CiproCl and NaCipro but deviates slightly in Cipro­(z), likely due to minor adjustments in electronic distribution.

**Table 1:**
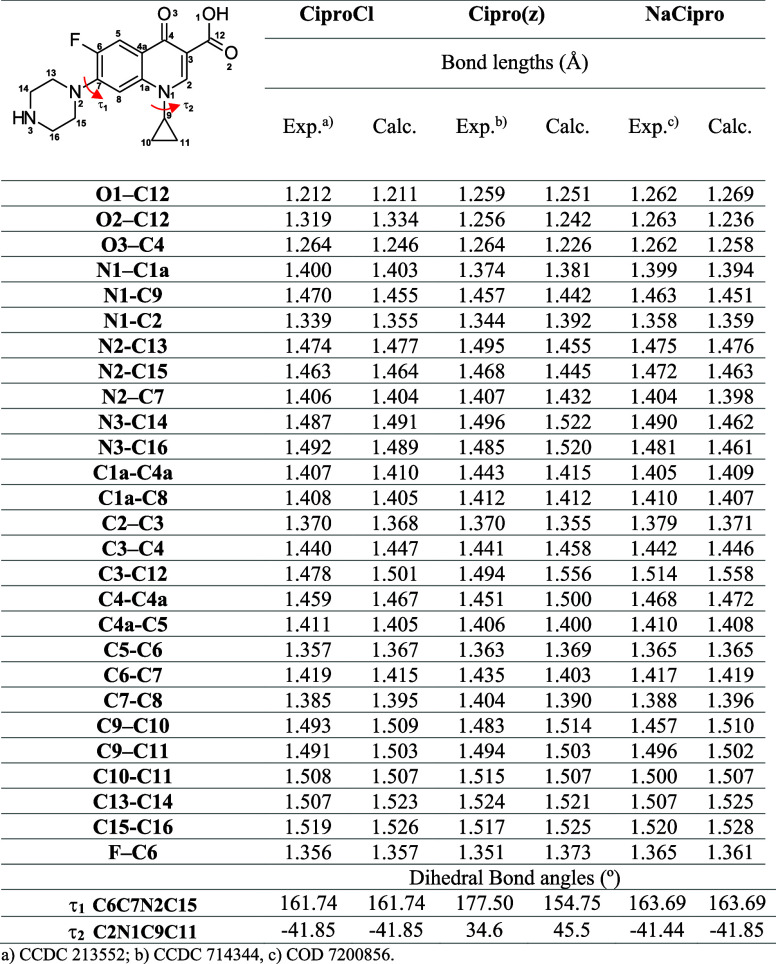
Bond Lengths (Å) and Torsion Angles (deg) for CiproCl, Cipro­(z), and NaCipro, Calculated at the B3LYP/6-311G­(d,p) Level of Theory

a CCDC 213552.

b CCDC 714344.

c COD 7200856.

Simulating the spectroscopic properties of a molecular crystal may be computationally prohibitive. Hence, firstly, it was necessary to evaluate the gas-phase description of the molecule to verify whether it preserves the intramolecular organization. This modeling strategy, which focuses on isolated molecules, is consistent with well-established approaches in the literature.^43–45^ Given the overall agreement between bond lengths and bond angles (Table 1 and Figure 5), the structures obtained from the gas-phase simulation were subsequently used to calculate the vibrational and ^13^C-NMR spectra.

### Sample Characterization by Vibrational Spectroscopy

A comparison among the three vibrational spectral profiles revealed some significant modifications related to the protonation level of the Cipro species in the solid state (Figures S6 and S7), as highlighted by the gray bars in Figure 6. Similar variations were observed in the IR spectra of Cipro in aqueous solutions in the pH range of 2 to 9.^24,25^ The band at about 1700 cm^–1^ is present only at CiproCl spectra and was attributed in several studies to the stretching C=O mode of the carboxyl group (Figure 6).^35,46,47^ The shoulder at 1609 cm^–1^ in the protonated form seems to be shifted to about 1587–1584 cm^–1^ after the carboxyl group deprotonation, as shown in Cipro­(z) and NaCipro spectra. The band at 1518 cm^–1^ of CiproCl loses intensity when compared to the same region in the Cipro­(z) and NaCipro infrared spectra, as well as the band at about 1447 cm^–1^ (Figure 6). On the other hand, the spectra of CiproCl did not show a band at approximately 1364–1361 cm^–1^ (see the Raman spectrum) and also at 1298–1284 cm^–1^ (see the infrared spectrum), which are observed in the Cipro­(z) and NaCipro spectra. Hence, these six mentioned spectral regions can be used to identify Cipro species in distinct protonation levels when free or associated with diverse materials, such as drug carriers or minerals in the environment.

**Figure 6:**
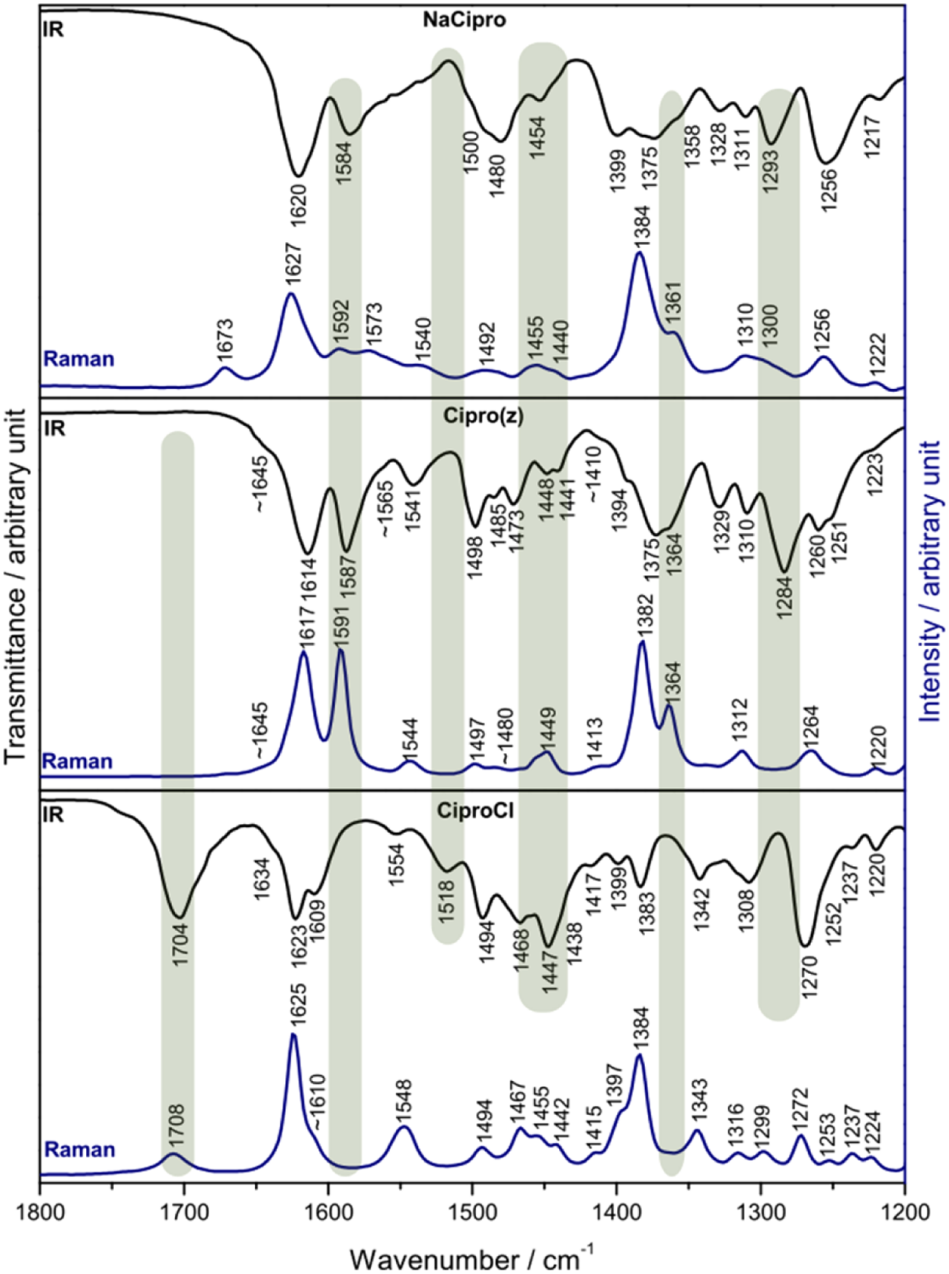
Experimental vibrational (infrared and Raman) spectra of NaCipro, Cipro­(z), and CiproCl samples.

To investigate the interactions among Cipro forms and other species (lipids, minerals, biomolecules, etc.), it is necessary to assign the vibrational bands to get information about the groups involved in the interfaces. However, the vibrational spectra of quinolones exhibit several bands that challenge the unequivocal assignment of vibrational modes. Although some computational calculations have been made to attribute the Cipro vibrational bands, the focus has not been on comparing the Cipro forms and fingerprinting the bands characteristics of each one. To shed light on the attribution of vibrational spectra of Cipro species, the infrared and Raman spectra of CiproCl, Cipro­(z), and NaCipro were simulated by the DFT method. The visual inspection of the calculated vibrational modes suggests that the experimental bands primarily involve different groups or atomic movements in the ciprofloxacin species. Some vibrational modes obtained by DFT calculations, to support the discussion in this section, are illustrated in Figures S8-S10.

Figure 7 shows the experimental and DFT-calculated infrared and Raman spectra of CiproCl, prepared in this work and characterized as mentioned earlier. The simulated spectral profiles are in good agreement with experimental spectra. The assignments of the most prominent vibrations, as shown in Table S2, enabled a correlation coefficient (R-squared, R^2^) of approximately 0.99 between the experimental and calculated wavenumbers (Figure S11), indicating a strong and excellent fit.

**Figure 7:**
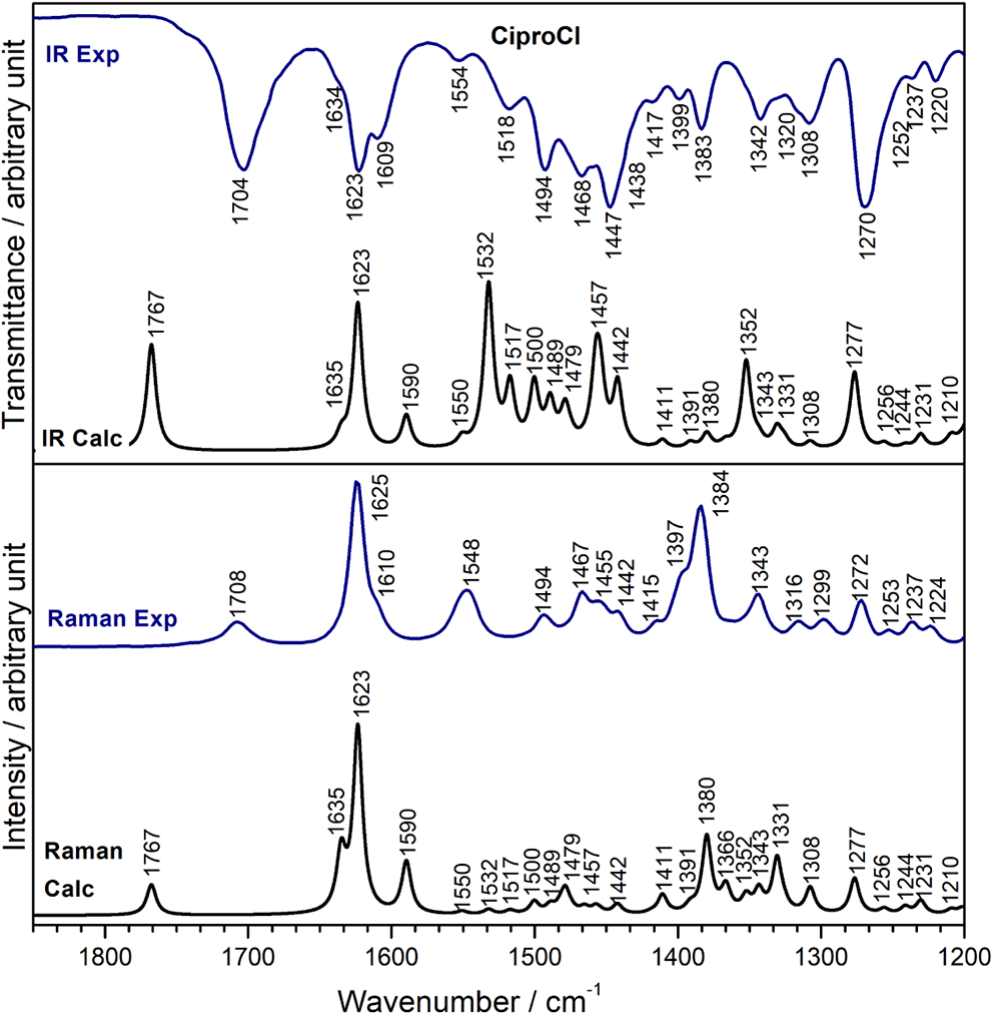
Experimental (Exp) and DFT-calculated (Calc) infrared and Raman spectra of CiproCl.

Notably, the band at 1767 cm^–1^, attributed to νC=O carboxyl + δipOH vibrational modes, showed no close correlation with the experimental band (1704–1708 cm^–1^). X-ray structural data showed that the carboxyl group of CiproCl is involved in hydrogen bonds with water molecules present in the crystal.^12^ DFT simulations of CiproCl spectra were performed in a vacuum, which explains the band’s higher wavelength when compared to the experimental value. DFT calculations were used in a supportive and qualitative manner, and spectral assignments based on theoretical data should be interpreted with caution, particularly in regions significantly influenced by solid-state effects.

Figure 8 presents the experimental and calculated vibrational spectra of Cipro­(z). The tentative assignments are shown in Table 2. A correlation coefficient R^2^ equal to 0.99 was observed between the experimental and calculated results (Figure S12). As observed for the protonated species, the band calculated at 1676 cm^–1^ for Cipro­(z), assigned to νC=O (pyridone) + ν_as_COO^–^ + νCC (aromatic), is observed with very low intensity at approximately 1645 cm^–1^ (Figure 8). Crystallographic data of anhydrous Cipro­(z) showed that the molecules are joined as dimers interacting electrostatically through the negative carboxylate group (−COO^–^) and the positive piperazinium moiety (NH_2_^+^),^5^ which can explain the experimental shift compared to the calculated value in a vacuum.

**Figure 8:**
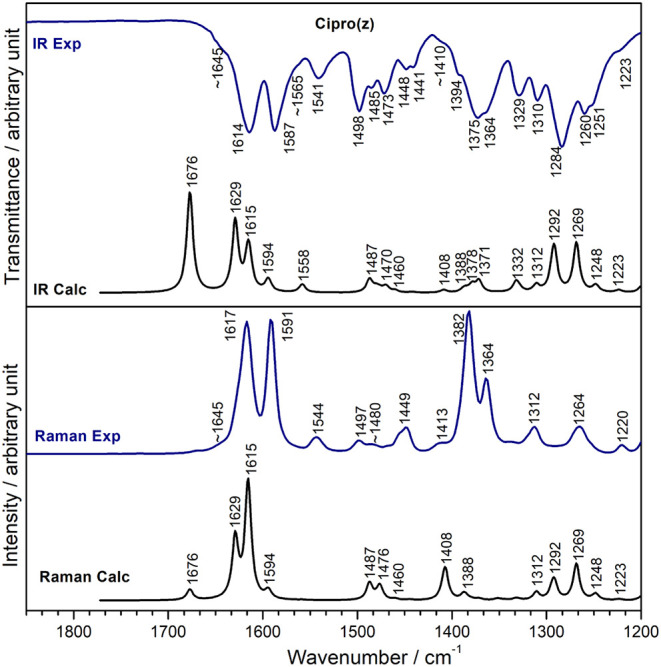
Experimental (Exp) and DFT-calculated (Calc) infrared and Raman spectra of Cipro­(z).

**Table 2:** Experimental Solid-State ^13^C CPMAS NMR Chemical Shift (ppm) and Calculated Chemical Shifts for CiproCl, Cipro­(z), and NaCipro^a^

	CiproCl		Cipro(z)			NaCipro			
	^13^C (ppm)								
	Label	Calc.	Exp.	Label	Calc.	Exp.	Label	Calc.	Exp.
Cyclo propyl	C11	8.29	6.8	C11	8.63	7.72^a^	C11	8.12	9.17^a^
	C10	10.6	9.7	C10	9.96	7.72^a^	C10	10.3	9.17^a^
	C9	36.5	34.4	C9	33.6	35.6	C9	36.1	37.2
Piperazine	C15	46.2	44.0^a^	C15	49.4	42.6	C16	49.6	44.6^a^
	C16	46.6	44.0^a^	C16	52.4	45.9^a^	C14	49.9	44.6^a^
	C14	47.3	47.4	C13	52.4	45.9^a^	C15	51.6	44.6
	C13	48.0	49.2	C14	54.0	47.5	C13	53.5	49.2
Quinolone	C8	108	105	C8	103	106	C8	106	105
	C3	116	111	C5	122	116	C5	119	109
	C5	119	121	C3	136	120	C3	126	111
	C4a	129	137	C7	137	123	C4a	129	118
	C1a	144	144	C4a	138	138	C1a	143	139
	C7	150	148	C1a	144	142	C7	149	145
	C2	152	150	C2	151	143	C2	156	148
	C6	161	151	C6	157	151	C6	161	151
			154			154			155
	C12	168	171	C12	162	173^a^	C12	167	168
	C4	180	175	C4	174	173^a^	C4	182	176

a Unresolved peaks.

The experimental and calculated vibrational spectra of NaCipro are presented in Figure 9. The corresponding correlation coefficient R^2^ (Figure S13) was also approximately 0.99, considering the 1700–1200 cm^–1^ range. These results suggest that the arrangement proposed for the DFT simulation of NaCipro is analogous to the Cipro^–^ and Na^+^ array in the solid isolated in this work.

**Figure 9:**
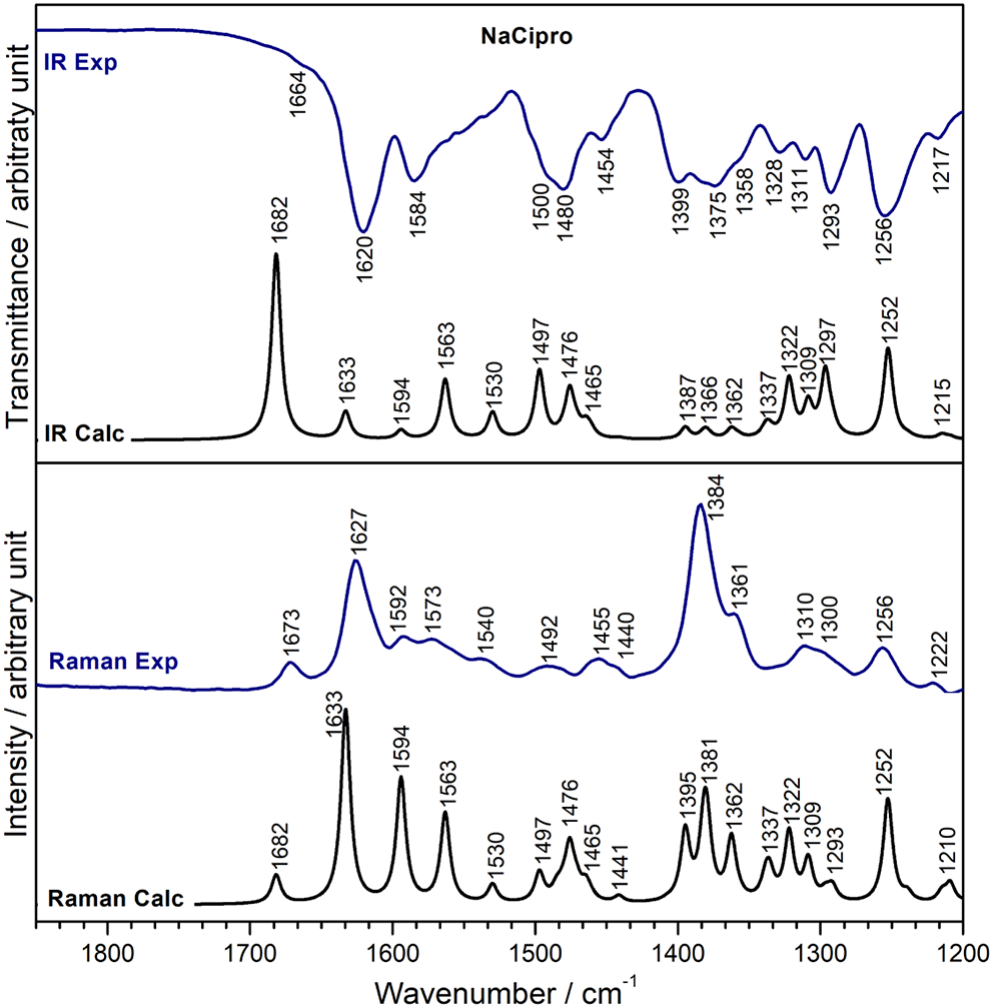
Experimental (Exp) and DFT-calculated (Calc) infrared and Raman spectra of NaCipro.

The band assignments of the six main spectral differences among the CiproCl, Cipro­(z), and NaCipro forms are reported and highlighted in Table S2. The band at 1704 cm^–1^, assigned mainly to νC=O carboxyl + δipOH vibrational modes (Figure 6), is present only in the CiproCl sample. Bands in the 1630–1550 cm^–1^ range are related to νCC in aromatic and pyridone rings associated with other vibrational modes (Table S2), which promote modifications in the spectra of cipro forms. The band at 1609 cm^–1^ of CiproCl is attributed to νC=O pyridone + δipOH, in addition to the aromatic νCC (Figure 6). Hence, it is expected to shift when Cipro is deprotonated and, indeed, the band appears at 1573 cm^–1^ in the NaCipro spectrum.

In the case of Cipro­(z) and NaCipro, the band at 1591–1565 cm^–1^ (Figure 6) is assigned to C=C stretching modes combined with scNH_2_^+^, and the band at 1592/1584 cm^–1^ is assigned to C=C stretching modes associated with ν_as_COO^–^, respectively. The band at 1518 cm^–1^, displayed only in the CiproCl infrared spectrum, is attributed to scNH_2_^+^ mode. Despite the presence of weak bands related to CH and CH_2_ deformation modes around 1440 cm^–1^ in all Cipro forms (Table S2), the strong band at 1447 cm^–1^, related to δipOH (Figure 6, IR spectrum), can be used as a fingerprint of CiproCl.

The bands at 1364 and 1361 cm^–1^ in the Raman spectra of Cipro (z) and NaCipro, respectively, are not observed in the spectra of the protonated form (Figure 6). These bands are primarily assigned to deformations of the CH and CH2 groups of the cyclopropyl ring (Table S2). Another significant change occurred in the 1300–1280 cm^–1^ range of the infrared spectra (Figure 6): the zwitterion and anionic forms showed bands related to the νsCOO^–^ mode at 1284 and 1293 cm^–1^, respectively, which are absent in the CiproCl spectra.

Besides the six vibrational regions mentioned above as signatures of the ciprofloxacin protonation level, other vibrational bands were also attributed as follows. Although the stretching of the C=O (pyridone) mode is always combined with other motions for all three ciprofloxacin forms, DFT calculations indicated that this vibrational mode is related to the bands at 1634 (weak), 1623–1625 and 1609 cm^–1^ for CiproCl, 1645 (weak) and 1614–1617 cm^–1^ for Cipro­(z), and 1540 cm^–1^ for NaCipro (Figure 6and Table S2). The red shift of carbonyl (pyridone) bands of NaCipro could be due to the ion-dipole interaction C=O···Na^+^ (Figure S10) that decreases the bond order of the carbonyl group to a greater extent than in the other forms. Indeed, the bond lengths of the C=O (pyridone) bonds calculated were 1.226, 1.246, and 1.258 Å for Cipro­(z), CiproCl, and NaCipro, respectively (Table 1).

Very few studies mention the NH_2_^+^ group in the vibrational attribution of CiproCl or Cipro­(z). Bands associated with the scissoring deformation of the NH_2_^+^ group in the piperazinum ring were observed at higher wavenumbers for Cipro­(z) (1614–1565 cm^–1^) than for CiproCl (1518–1455 cm^–1^). According to crystal data, the protonated amine group is involved in NH_2_^+^···^–^OOC interaction in the Cipro­(z). In CiproCl, the NH_2_^+^···Cl^–^ interaction is observed, and it can be stronger, making the NH_2_^+^deformation difficult.

The antisymmetric −COO^–^ stretching mode of Cipro­(z) and NaCipro contributes, respectively, to the weak bands at about 1645 and 1673 cm^–1^, and the strong bands at 1614–1617 cm^–1^ and 1584–1592 cm^–1^ (Figure 6 and Table S2). On the other hand, the symmetric −COO^–^ stretching mode is coupled with other vibrational modes and assigned to 1284 and 1260 cm^–1^ (zwitterion), 1311 and 1293 cm^–1^ (NaCipro). Symmetric carboxylate stretching mode has been assigned in the literature to bands at 1380–1405 cm^–1^ .^6,24,48^ However, the strong bands at about 1384 cm^–1^ of all ciprofloxacin species, including the protonated CiproCl (Figure 6), were assigned in this work mainly to the deformation of −CH_2_ and −CH from pyridone and aromatic groups (Table S2), which seems reasonable. According to DFT simulations, bands of very low intensities below 1200 cm^–1^ (Figures S6 and S7) were assigned to C-F stretching mode (1165–1150 cm^–1^), twisting NH_2_^+^ (1141 cm^–1^), δCOO-H (855–852 cm^–1^), δopCOO^–^ (800–810 cm^–1^), and δipCOO^–^ (689 cm^–1^).

The vibrational spectrum of an anionic salt (in the solid state) of ciprofloxacin was presented for the first time in this work, along with its DFT-simulated spectra. Theoretical work has been reported on metal-ciprofloxacin complexes (including sodium), but none of the proposed forms is related to the one observed for the NaCipro sample. It is worth mentioning that Bodo et al.^34^ calculated the infrared vibrational spectra of neutral Cipro, Cipro­(z), and protonated Cipro in the gas phase and compared them with the infrared multiple photon dissociation spectra of the drug species in the gas phase generated by electrospray of a methanol solution. The vibrational spectrum of Cipro­(z) was calculated but not assigned. The experimental spectrum was better related to that one calculated for protonated Cipro, being that the following bands were assigned^34^ (exp./calc.): νC=O carboxyl (1762/1771 cm^–1^), νC=O pyridone (about 1640/1637 cm^–1^), νCC aromatic (about 1600/1618 cm^–1^), NH_2_^+^ deformation (about 1585/1595 cm^–1^), δipCH (1467/1486 cm^–1^), δOH (1389/1427 cm^–1^), rocking CH_2_ of piperazinium (1255/1258 cm^–1^). These attributions do not agree with those shown here in Table S2 for CiproCl, but it is necessary to consider that samples are distinct: protonated Cipro at the gas state^34^ and protonated Cipro at the solid state with a counter ion (CiproCl). However, the stretching of the C=O carboxyl group, as shown in the experimental and calculated spectra of the gas species, is very close to the value calculated in this work for CiproCl in vacuum (1767 cm^–1^). Hence, the assumption that this band is redshifted to 1704 cm^–1^ (Figure 6 and Table S2) due to intramolecular hydrogen bonds in a solid state is quite plausible.

### Sample Characterization by Solid-State ^13^C NMR Spectroscopy

The ^13^C solid-state NMR spectra, theoretical NMR chemical shifts, and the corresponding numerical values for the Cipro forms are presented in Figure 10 and Table 2. The DFT simulations were primarily used to support the assignments of specific chemical environments, ensuring precise correlation between experimental observations and molecular electronic effects. On average, the obtained theoretical deviations were around 1.5 ppm, 4.5 ppm, and 5.5 ppm for the cyclopropyl group, piperazine ring, and quinolone core, respectively. Correlation coefficients R^2^ higher than 0.99 were observed between the experimental and calculated results for CiproCl, Cipro­(z), and NaCipro (Figures S14-S16). The deshielding variations induced by protonation and counterion interactions differentiate the three ciprofloxacin forms, with the quinolone core (C1a–C8, C12) and piperazine moiety (C13–C16) being the most responsive to charge redistribution and electronic effects.

**Figure 10:**
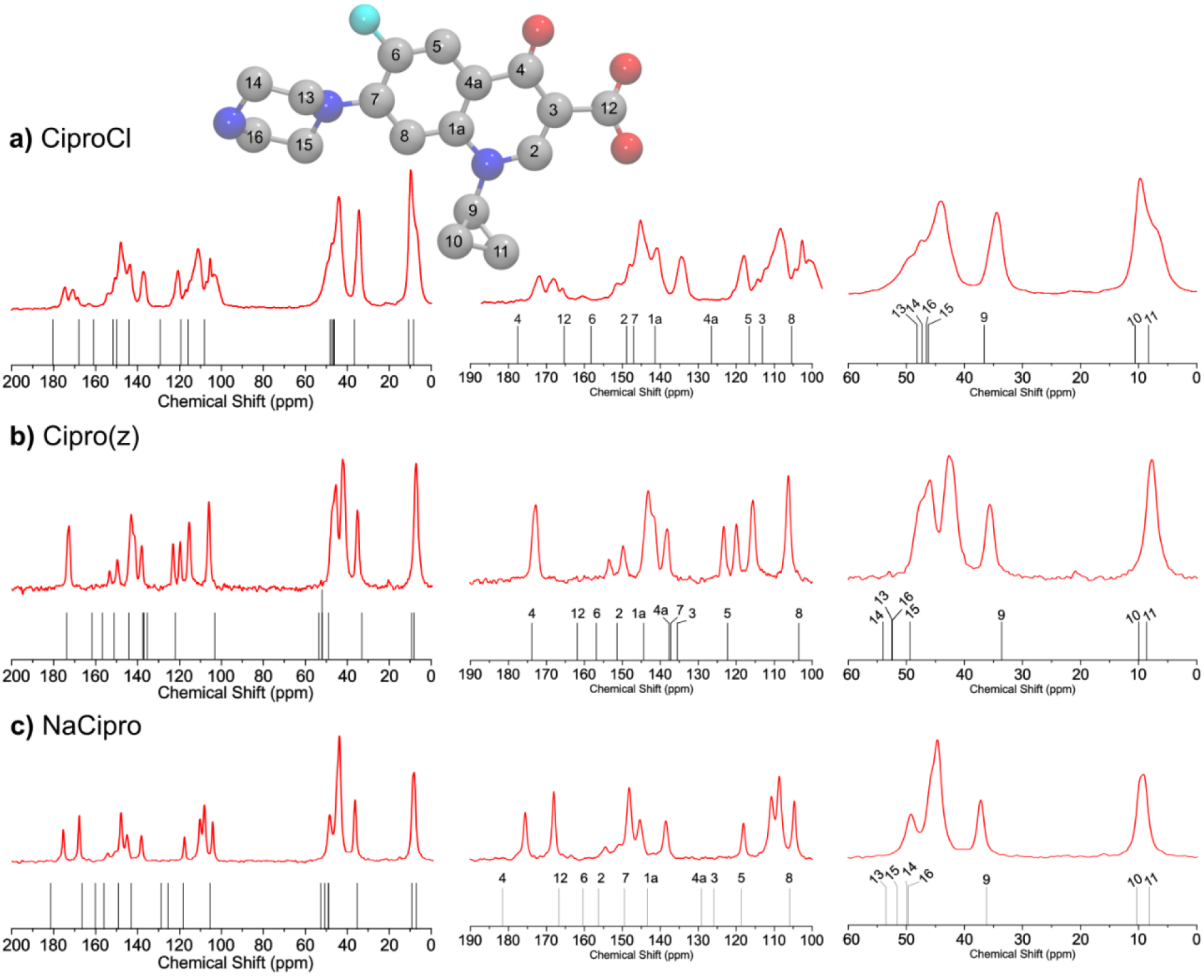
^13^C CP-MAS solid-state spectra (red line) and theoretical NMR chemical shifts (black ticks) obtained for (a) CiproCl, (b) Cipro­(z), and (c) NaCipro. Insert: molecule with atom numbering (H atoms were hidden). Atomic color: gray (C), red (O), blue (N), Cyan (F).

The quinolone core carbons (C1a–C8, C12) exhibited moderate but systematic chemical shift variations, reflecting the redistribution of electronic density across the aromatic system. A doublet (150 and 154 ppm), well resolved for Cipro­(z), is observed due to the spin-spin coupling between the C6 and the fluorine atoms.^4^ In contrast to CiproCl and NaCipro spectra that showed distinct C12 and C4 peaks (170–180 ppm, Figure 10), Cipro­(z) presented only one peak. The amplification of this spectral region indicated the overlap of unresolved peaks that can be assigned to the carboxylate and pyridone C=O groups. Mafra et al.^4^ assigned signals at 172.7 and 173.1 ppm to C12 and C4, respectively.

The C2 carbon (143–151 ppm) also showed noticeable variations (Δδ around 8 ppm), which can be attributed to electronic effects from the adjacent carbonyl groups (C4 and C12). Additional structural differences were observed for C1a (138–144 ppm) and C4a (118–137 ppm), whose shifts reflect global electronic redistribution within the aromatic ring. The more pronounced shift of C4a in NaCipro (Δδ = −20 ppm relative to Cipro­(z)) suggests that charge redistribution in the deprotonated form extends further into the quinolone system, altering conjugation patterns.

The piperazine ring carbons (C13–C16) display moderate chemical shift variations, primarily reflecting localized electronic effects. In CiproCl and Cipro­(z), the nitrogen remains protonated (NH_2_^+^), leading to slight deshielding of adjacent carbons (C14 and C16). In contrast, in NaCipro, the loss of this positive charge results in a slight but consistent upfield shift across the ring. These results confirm that while the piperazine moiety is affected by protonation, its impact on the overall electronic environment is more localized compared to the quinolone conjugated core (sp^2^ hybridization in quinolone versus sp^3^ hybridization in piperazine) that carries many chemical functions capable of interacting and thus propagating the effect through the entire quinolone backbone.

Finally, the cyclopropyl group (C9–C11) is the least affected by protonation and counterion interactions, displaying minimal chemical shift variations (Δδ < 3 ppm). The most significant variation is observed for C9 (34.4–37.2 ppm), while C11 exhibits a small deshielding effect (6.8–9.17 ppm). These results confirm that the cyclopropyl moiety is electronically stable, reinforcing its role as a rigid structural substituent rather than an electronic probe for differentiating protonation states.

### Molecular Electrostatic Potential (MEP) Surface

Figure 11 displays the electrostatic potential maps for CiproCl, Cipro­(z), and NaCipro. In these maps, red indicates the most negative potential, blue represents the most positive potential, and green denotes regions of zero potential. Cipro­(z) exhibits the highest charge separation, characterized by a more pronounced positive potential than CiproCl and a more significant negative potential than NaCipro. The positive regions are associated with the piperazinium group and the sodium ion. In contrast, the negative regions are located around the carbonyl and carboxylate groups, as well as the chloride ion. This information can be useful for analyzing the Cipro form’s interaction with surfaces in future studies.

**Figure 11:**
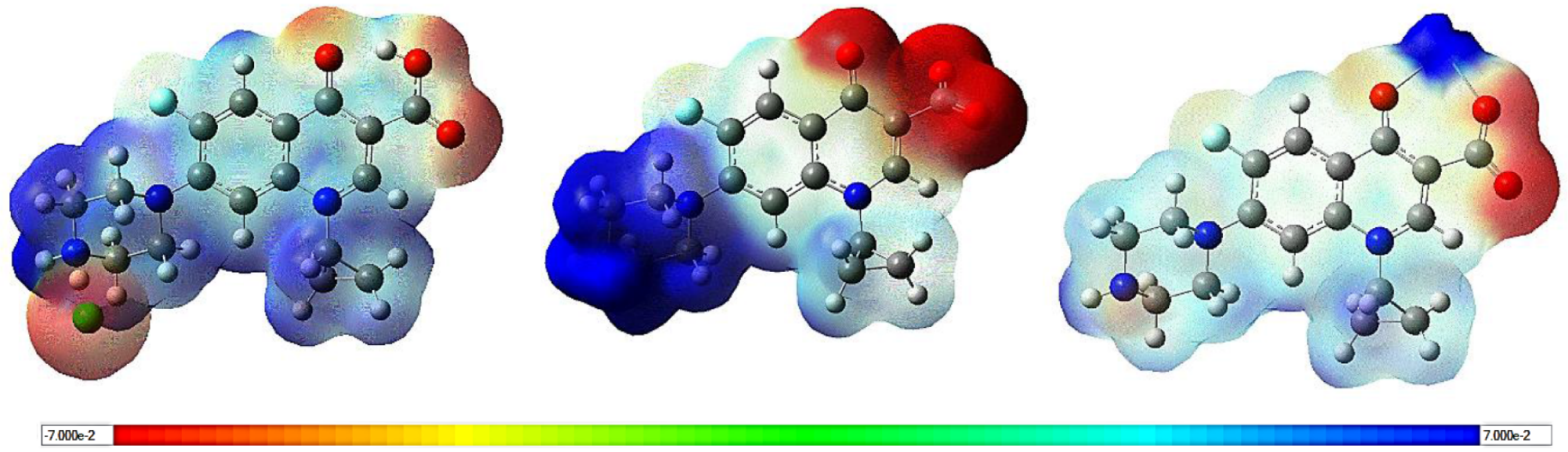
Molecular electrostatic potential maps of CiproCl (left), Cipro­(z) (middle), and NaCipro (right) obtained from DFT calculations.

## Conclusions

The structural and spectroscopic analyses provided a thorough characterization of ciprofloxacin in its zwitterionic, cationic, and anionic forms. X-ray diffraction (XRD) patterns confirmed the crystallinity of all samples, with Cipro­(z) and CiproCl displaying patterns consistent with monocrystalline structures. In contrast, NaCipro exhibited a distinctive crystalline pattern that differed from previously described sodium ciprofloxacin salts. Thermal analysis revealed variations in hydration and thermal stability among the ciprofloxacin forms, particularly for NaCipro, whose data indicated that it was obtained as a monohydrate salt, and its decomposition led to NaF as the residue. Vibrational spectroscopy identified several spectral features that serve as markers of ciprofloxacin’s protonation states: (*i*) 1700 (carboxyl stretching and OH deformation); (*ii*) 1600–1580 (C=C stretching associated to carboxylate antisymmetric stretching); (*iii*) 1520 (scissoring deformation of NH_2_^+^); (*iv*) 1470–1440 (OH deformation associated to NH_2_^+^ and CH_2_ wagging modes in piperazinium); (*v*) 1360 (deformations of CH and CH_2_ of the cyclopropyl group); and (*vi*) 1300–1280 cm^–1^ (carboxylate symmetric stretching). ^13^C CP-MAS NMR spectroscopy provided additional evidence of electronic redistribution due to protonation and counterion interactions. The quinolone core and piperazine ring displayed systematic deshielding effects. Theoretical calculations supported these trends, reinforcing the electronic effects induced by protonation and sodium coordination. These results contribute to a comprehensive understanding of ciprofloxacin’s structural and spectroscopic properties, providing valuable reference data for pharmaceutical formulations and environmental studies.

## Supplementary Material



## Notes

Ciprofloxacin aqueous speciation in the pH range from 0 to 14 (Figure S1); XRD patterns of Cipro­(z), CiproCl, and NaCipro (Figure S2); XRD pattern of Cipro­(z) from Aldrich and simulated XRD pattern (Figure S3); XRD pattern of CiproCl obtained in this work and simulated XRD pattern (Figure S4); XRD pattern of NaCipro obtained in this work and simulated XRD pattern of NaCipro penta hydrate (Figure S5), FTIR spectra of Ciprofloxacin forms in the 4000–450 cm^–1^ range (Figure S6); Raman spectra of Ciprofloxacin forms in the 3500–200 cm^–1^ range (excitation laser = 1064 nm) (Figure S7); some vibrational modes of Cipro­(z), CiproCl, and NaCipro, obtained by DFT calculations (Figures S8–S10); correlation between experimental and calculated (DFT method) wavenumbers for Cipro­(z), CiproCl, and NaCipro vibrational bands (Figures S11-S13); correlation between experimental and calculated (DFT method); correlation between experimental and calculated (DFT method) ^13^C-NMR chemical shift for Cipro­(z), CiproCl, and NaCipro (Figures S14-S16); steps proposed for NaCipro thermal decomposition under synthetic air conditions (Scheme S1); XRD data of Cipro­(z), CiproCl, and NaCipro: 2θ (λ = 1.54 Å) and d (interplane distances) values (Table S1); Raman and IR wavenumbers (in cm^–1^) of CiproCl, Cipro­(z), and NaCipro, calculated vibrational wavenumbers (in cm^–1^), and a tentative assignment (Table S2) (PDF)

## Notes

We dedicate this paper to our deceased co-author, Vanessa Yumi Sakai (former PhD student of Instituto de Química, Universidade de São Paulo, São Paulo—SP, Brazil).
