# Supplementary material for: Ciprofloxacin (Zwitterion, Chloride, and Sodium Forms): Experimental and DFT Characterization by Vibrational and Solid-State NMR Spectroscopies
Source: ACS Omega. 2025 Oct 11;10(41):48773–86. doi: 10.1021/acsomega.5c06384 (PMC12547570; doi:10.1021/acsomega.5c06384)
Supplement: Supplementary file 1 [file ao5c06384_si_001.pdf]

Supporting Information

Ciprofloxacin (Zwitterion, Chloride and Sodium forms): Experimental and DFT characterization by vibrational and solid-state NMR spectroscopies[§]

Filipe C. D. A. Lima[a], Arthur P. Camargo[b], Fabrice Leroux[c], Jocelyne M. Brendlé[d,e], Marcia L. A. Temperini[f], Helena M. Petrilli[b], Vera R.L. Constantino[f*]

[a]*Instituto Federal de Educação, Ciência e Tecnologia de São Paulo (IFSP), Campus Matão, Av. Stefano D'avassi, 625, CEP 15991-502 Matão - SP, Brazil.*
[b]*Departamento de Física dos Materiais e Mecânica, Instituto de Física, Universidade de São Paulo (USP), Rua do Matão, 1371, CEP 05508-090 São Paulo - SP, Brazil.*
[c]*Institut de Chimie de Clermont-Ferrand ICCF, CNRS UMR 6296, Université Clermont Auvergne, 24 avenue Blaise Pascal, F-63000 Clermont-Ferrand, France.*
[d]*Institut de Science des Matériaux de Mulhouse, CNRS UMR 7361, Université de Haute-Alsace, 15 rue Jean Starcky, F-68100 Mulhouse, France.*
[e]*Université de Strasbourg, 20A Rue René Descartes, F-67081 Strasbourg, France.*
[f]*Departamento de Química Fundamental, Instituto de Química, Universidade de São Paulo (USP), Av. Prof. Lineu Prestes 748, CEP 05508-000 São Paulo - SP, Brazil.*

[§] We dedicate this paper to our deceased co-author, Vanessa Yumi Sakai (former PhD student of Instituto de Química, Universidade de São Paulo, São Paulo - SP, Brazil).

***Corresponding author:** vrlconst@iq.usp.br

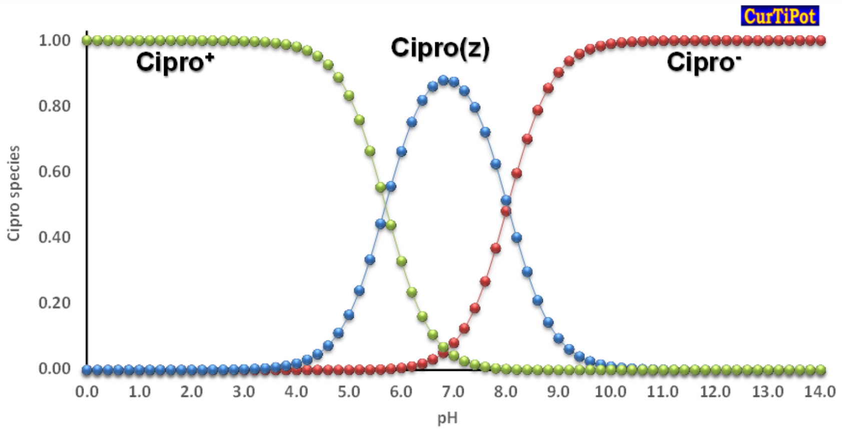

Fig. S1. Ciprofloxacin aqueous speciation in the pH range from 0 to 14.

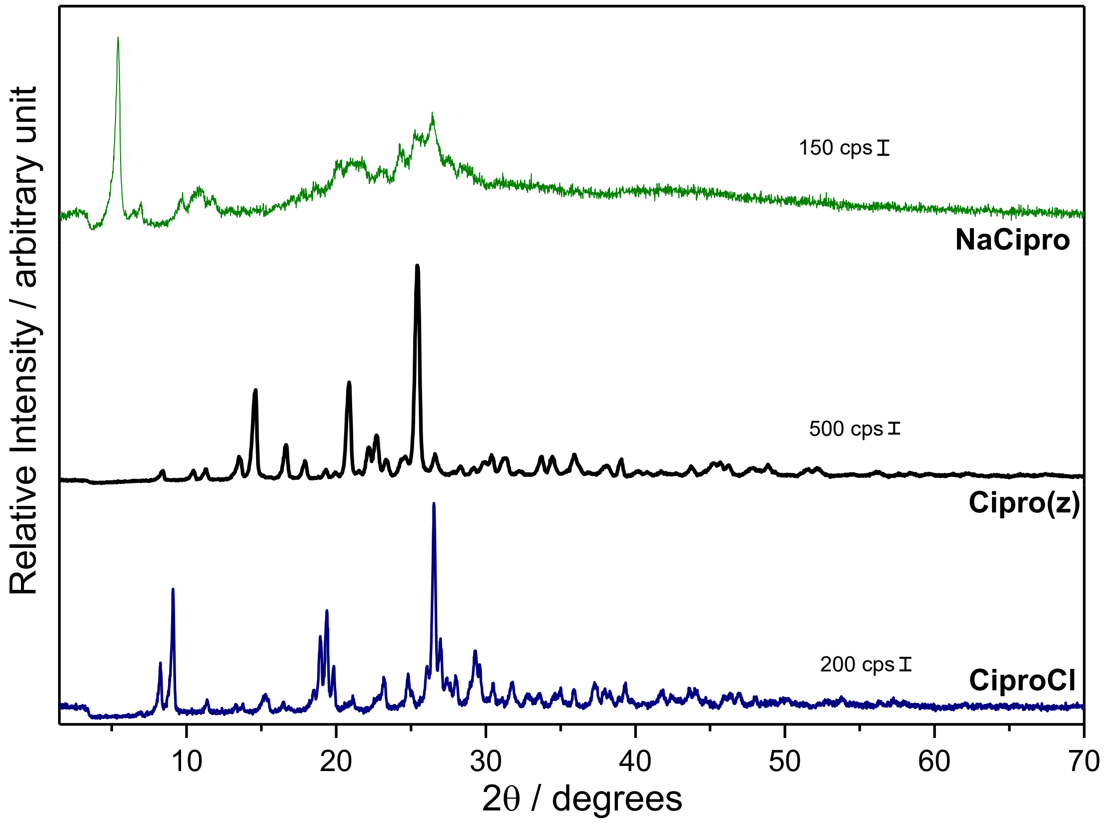

Fig. S2. XRD patterns of Cipro(z), CiproCl, and NaCipro.

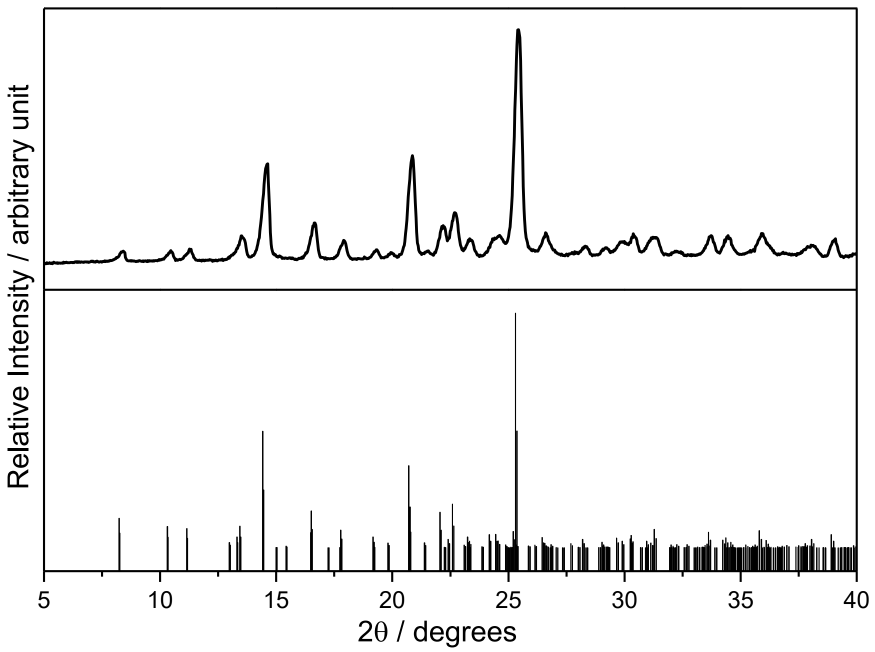

Fig. S3. XRD pattern of Cipro(z) from Aldrich (top) and simulated XRD pattern obtained from ref.[1] , using the Visualization for Electronic and Structural Analysis (VESTA) program (down).

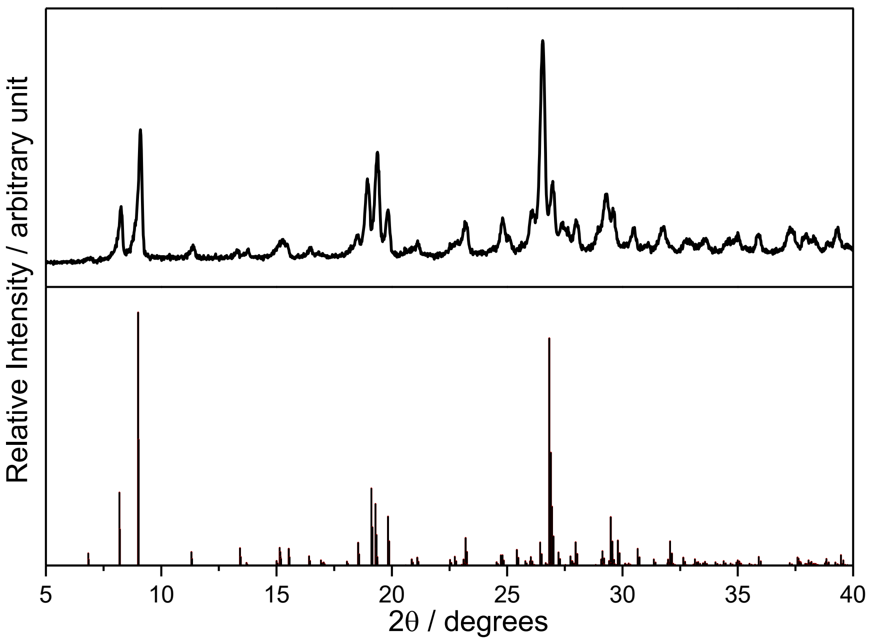

Fig. S4. XRD pattern of CiproCl obtained in this work (top) and simulated XRD pattern obtained from ref.[2] using the Visualization for Electronic and Structural Analysis (VESTA) program (down).

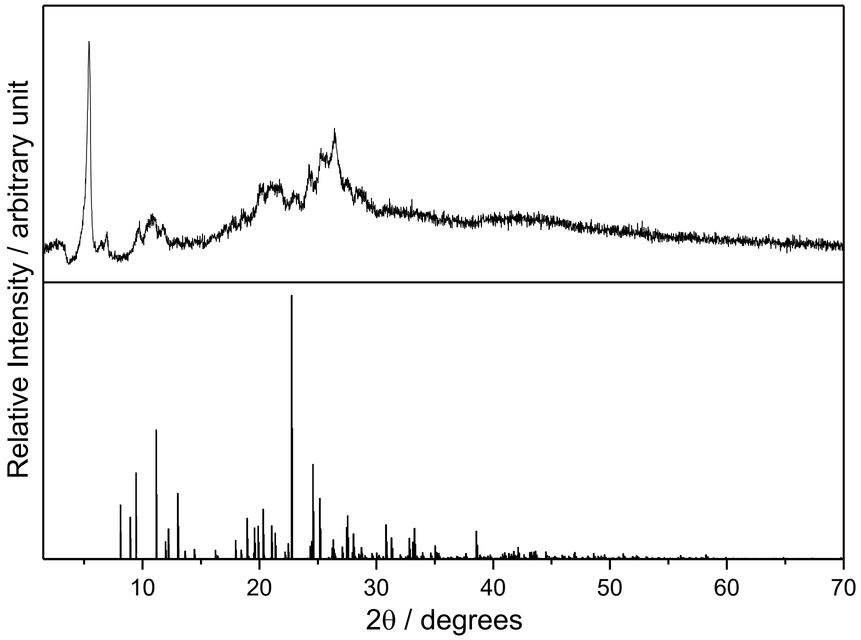

Fig. S5. XRD pattern of NaCipro obtained in this work (top) and simulated XRD pattern of NaCipro penta hydrate obtained from ref.[1], using the Visualization for Electronic and Structural Analysis (VESTA) program (down).

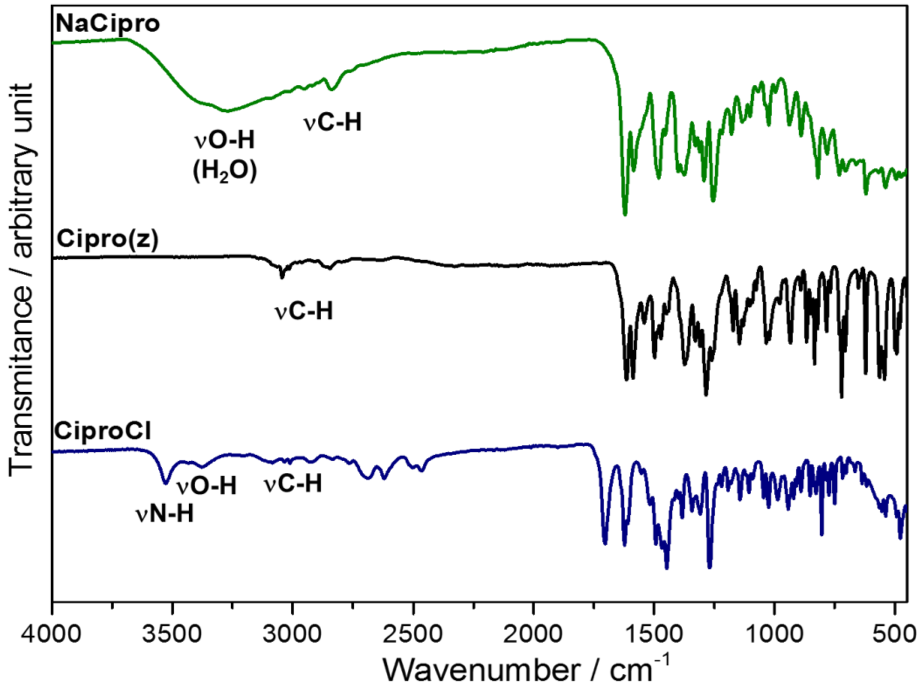

Fig. S6. FTIR spectra of Ciprofloxacin forms in the 4000 - 450 cm^{-1} range.

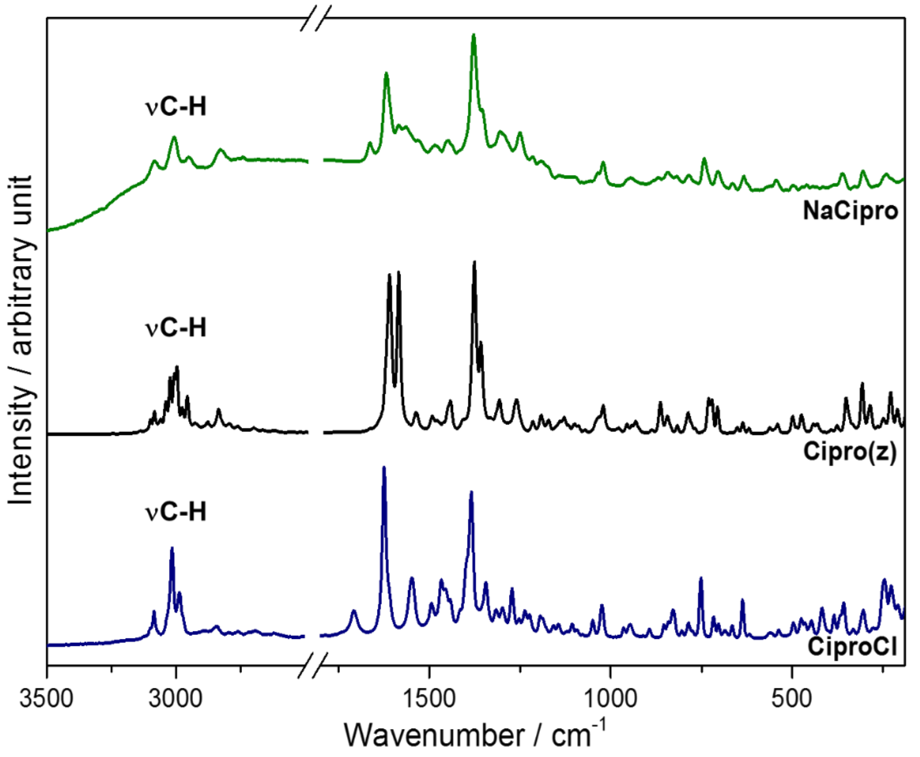

Fig. S7. Raman spectra of Ciprofloxacin forms in the 3500 -200 cm^{-1} range (excitation laser = 1064 nm).

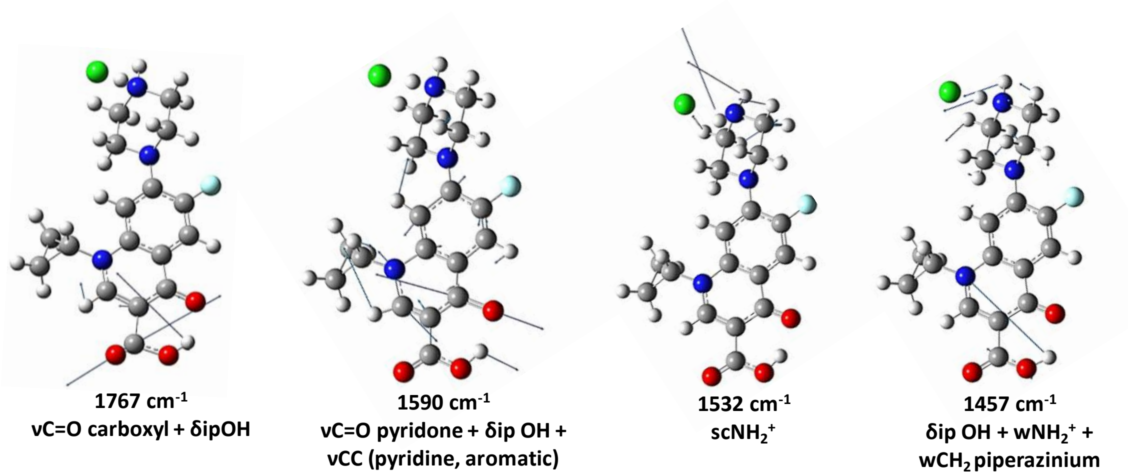

Figure S8. Some vibrational modes of CiproCl obtained by DFT calculations. Arrows show the direction of motion.

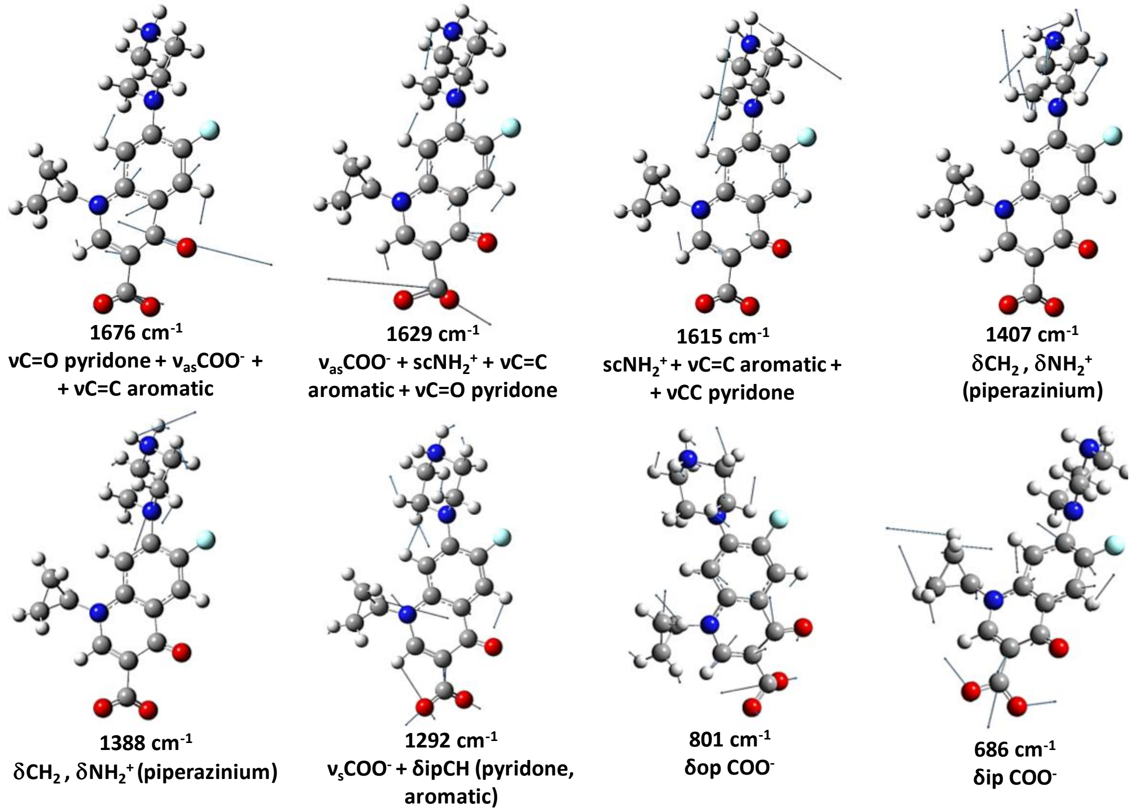

Figure S9. Some vibrational modes of Cipro(z) obtained by DFT calculations. Arrows show the direction of motion.

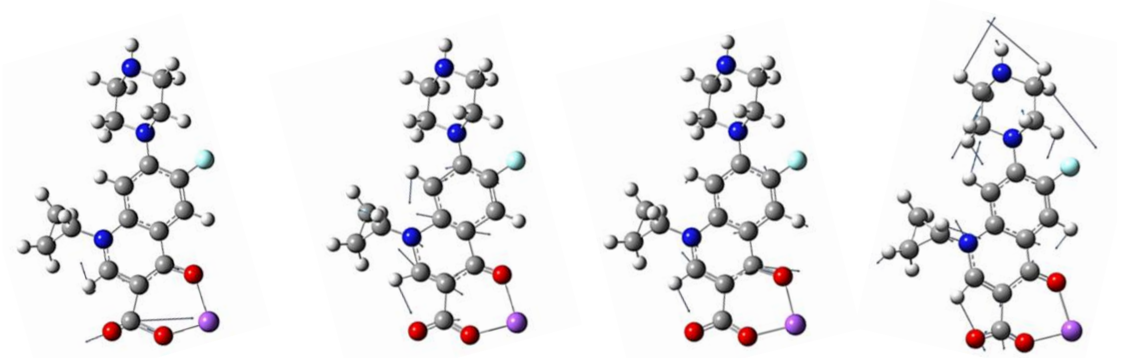

1682 cm^{-1}
$\nu_{as}COO^-$ + νC=O pyridone

1592 cm^{-1}
νCC (aromatic, pyridone) + $\nu_{as}COO^-$

1563 cm^{-1}
νC=O pyridone + νCC (pyridine, aromatic)

1297 cm^{-1}
tCH_2 piperazine + δipCH + $\nu_s COO^-$

Figure S10. Some vibrational modes of NaCipro obtained by DFT calculations. Arrows show the direction of motion.

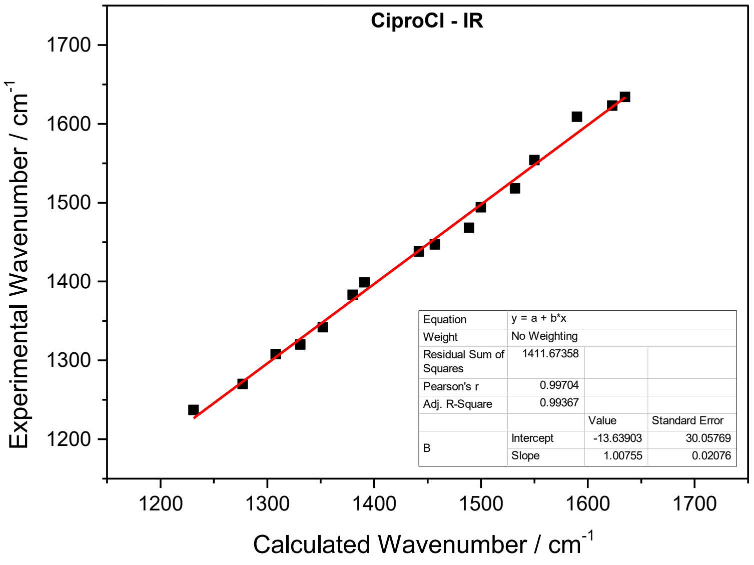

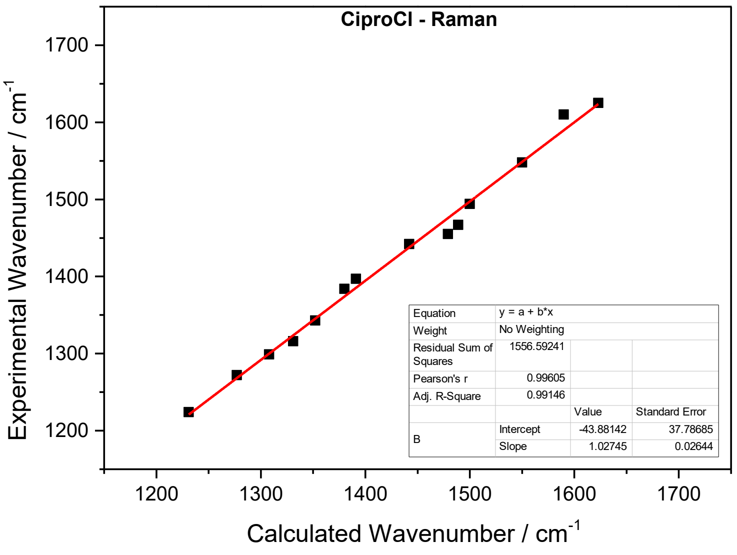

Fig. S11. Correlation between experimental and calculated (DFT method) wavenumbers for CiproCl bands, as listed in Table S2. The calculated band at 1767 cm^{-1} was not considered.

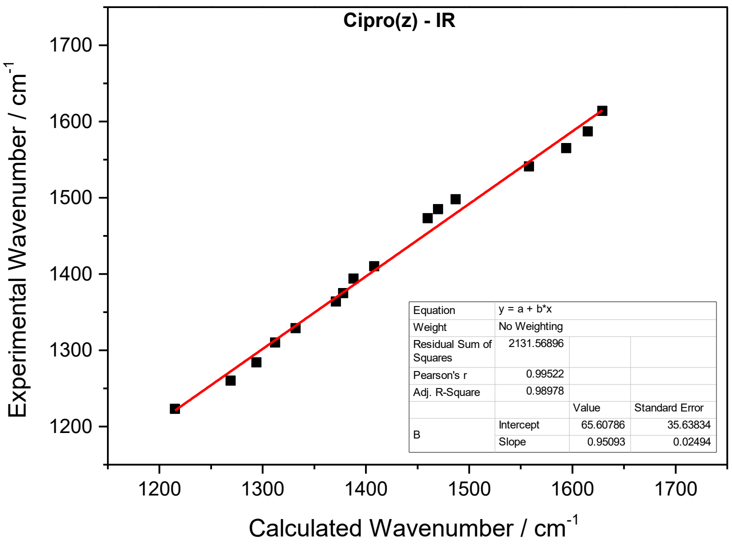

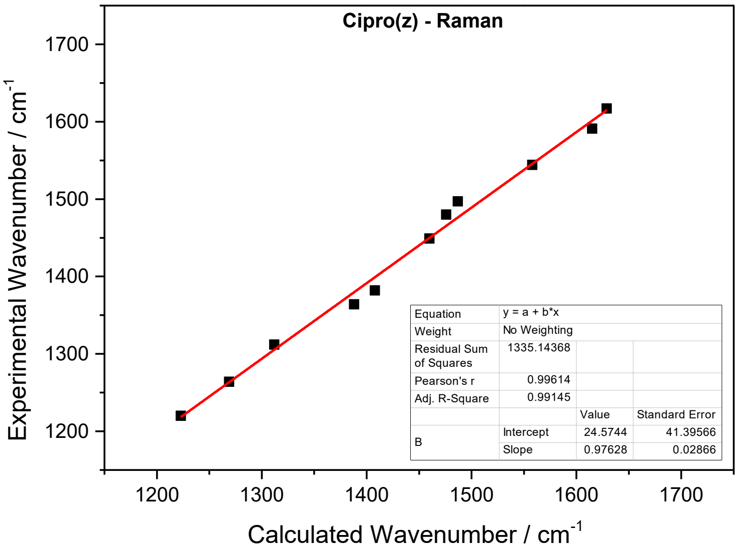

Fig. S12. Correlation between experimental and calculated (DFT method) wavenumbers for Cipro(z) bands, as listed in Table S2. The calculated band at 1676 cm^{-1} was not considered.

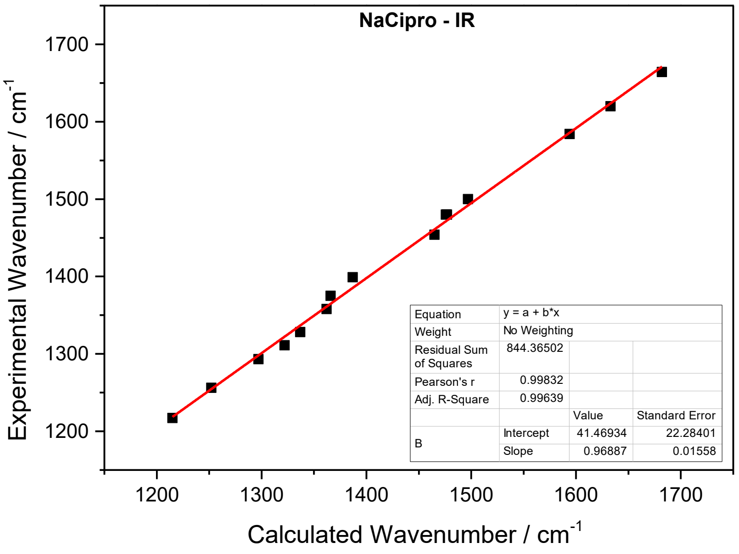

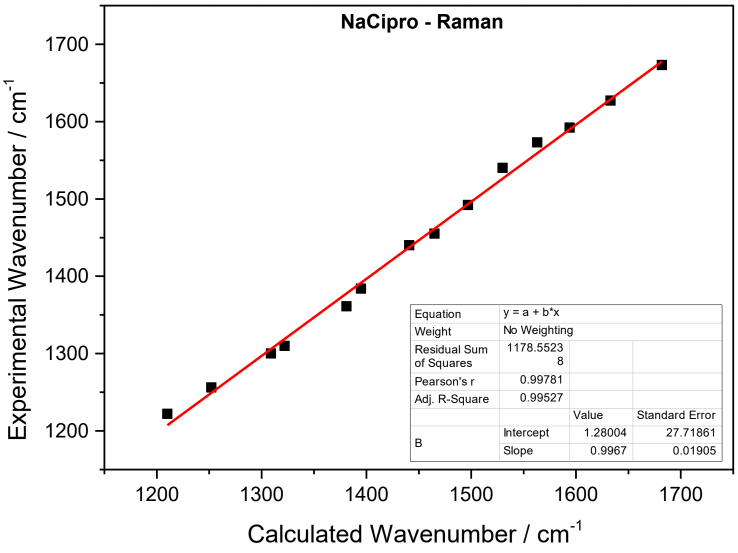

Fig. S13. Correlation between experimental and calculated (DFT method) wavenumbers for NaCipro bands, as listed in Table S2.

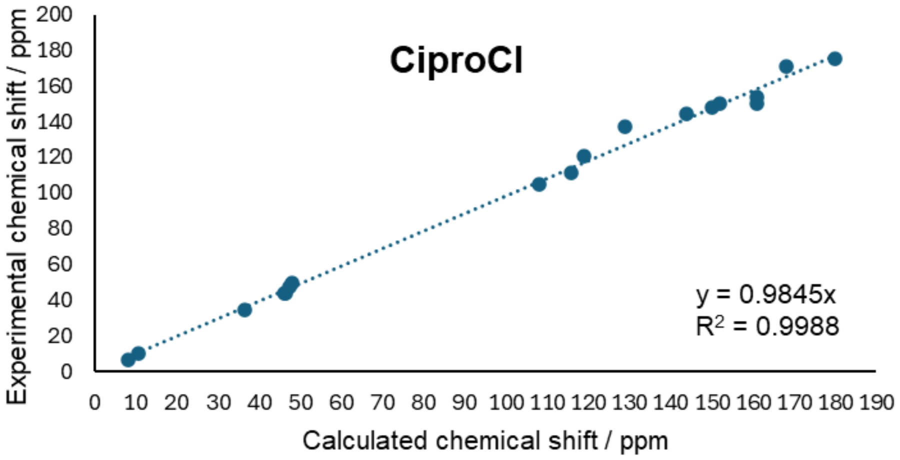

Fig. S14. Correlation between experimental and calculated (DFT method) ^{13}C-NMR chemical shift for CiproCl, as listed in Table 2.

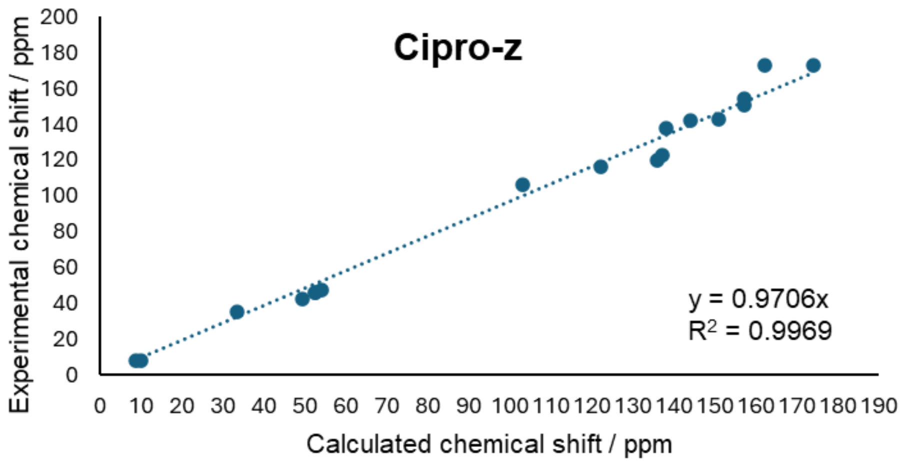

Fig. S15. Correlation between experimental and calculated (DFT method) ^{13}C-NMR chemical shift for Cipro(z), as listed in Table 2.

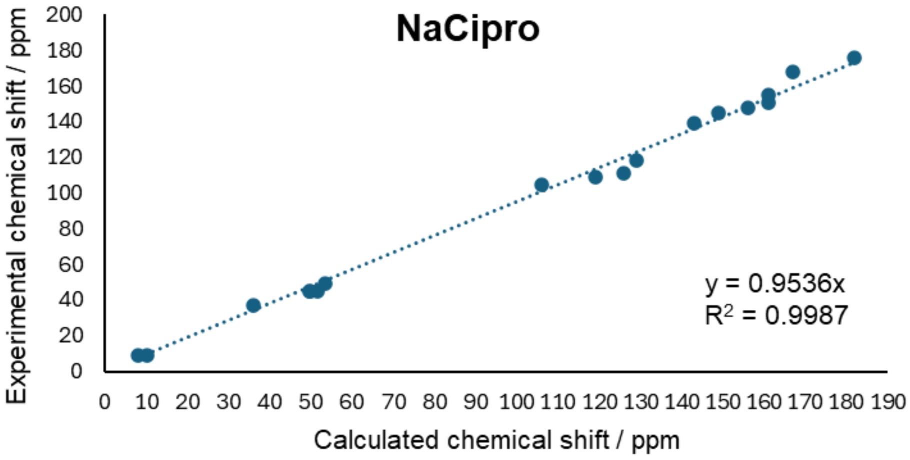

Fig. S16. Correlation between experimental and calculated (DFT method) ^{13}C-NMR chemical shift for NaCipro, as listed in Table 2.

Step 1: $Na(C_{17}H_{17}FN_3O_3)\cdot H_2O(s) \xrightarrow{\textit{up to 150ºC}} Na(C_{17}H_{17}FN_3O_3)(s) + H_2O$

Δm (calc) = 4.9% *Δm (exp) = 5%*

Step 2: $Na(C_{17}H_{17}FN_3O_3)(s) \xrightarrow{\textit{150-500ºC}} Na(C_{13}H_5FN_2)(s) + H_2O(g) + CO_2(g) + \{C_3NH_{10}\}(g)$

Δm (calc) = 32.9% *Δm (exp) = 33%*

Step 3: $Na(C_{13}H_5FN_2)(s) \xrightarrow{\textit{500-800ºC}} NaF(s) + \{C_{13}H_5N_2\}$

Δm (calc) = 50.9% *Δm (exp) = 50%*

residue (calc) = 11.3% residue (exp) = 12%

Scheme S1. Steps proposed for NaCipro thermal decomposition under synthetic air conditions.

Table S1. XRD data of Cipro(z), CiproCl, and NaCipro: 2θ (λ=1.54 Å) and d (interplane distances) values.

CiproCl			Cipro(z)			NaCipro	
**2θ / degrees**[1]	**2θ / degrees**	**d / Å Exp**	**2θ / degrees**[2]	**2θ / degrees**	**d / Å Exp**	**2θ / degrees**	**d / Å**
8.18	8.26	10.69	8.23	8.42	10.49	5.42	16.29
9.00	9.10	9.71	10.31	10.46	8.45	6.50	13.58
11.31	11.38	7.77	11.15	11.27	7.84	6.96	12.69
	15.28	5.79	13.43	13.49	6.56	9.74	9.07
18.54	18.54	4.78	14.42	14.63	6.05	10.86	8.14
19.11	18.94	4.68	16.51	16.67	5.31	11.76	7.52
19.29	19.38	4.57	17.78	17.90	4.95	20.36	4.36
19.83	19.84	4.47		19.31	4.59	20.96	4.23
23.29	23.16	3.84		19.94	4.45	21.72	4.09
	24.80	3.59	20.71	20.87	4.25	24.22	3.67
26.43	26.10	3.41		22.19	4.00	25.26	3.52
26.83	26.54	3.35		22.67	3.92	26.42	3.37
26.94	26.98	3.30	25.31	25.43	3.50	29.18	3.05
29.49	29.28	3.05		26.60	3.35		

Table S2. Raman and IR wavenumbers (in cm^{-1}) of CiproCl, Cipro(z), and NaCipro, calculated vibrational wavenumbers (in cm^{-1}), and a tentative assignment.

CiproCl			Cipro(z)			NaCipro			
**Calc.**[a)]	**Exp.**		**Calc.**[a)]	**Exp.**		**Calc.**[a)]	**Exp.**		**Assignment** (most prominent vibrations)[b)]
	**IR**	**Raman**		**IR**	**Raman**		**IR**	**Raman**	
**1767**	1704	1708							νC=O (carboxyl), δipOH
						**1682**	1664 (sh)	1673	ν_{as}COO$^-$, νCC (pyr)
			**1676**	1645 (sh)	1645 (sh)				νC=O (pyr), ν_{as}COO$^-$, νCC (arom)
**1635**	1634 (sh)								νCC (arom), νCC (pyr), νC=O (pyr)
						**1633**	1620	1627	νCC (arom), νCC (pyr)
			**1629**	1614	1617				ν_{as}COO$^-$, scNH$_2^+$, νCC (arom), νC=O (pyr)
**1623**	1623	1625							νC=O (pyr), νCC (pyr), νCC (arom), νC=O (carboxyl)
			**1615**	1587	1591				scNH$_2^+$, νCC (pyr), νCC (arom)
						**1592**	1584	1592	νCC (arom), νCC (pyr), ν_{as}COO$^-$
			**1594**	1565 (sh)	(sh)				scNH$_2^+$, νCC (pyr), νCC (arom)
**1590**	1609	1610 (sh)							νC=O (pyr), δipOH, νCC (pyr), νCC (arom)
						**1563**	(sh)	1573	νC=O (pyr), νCC (pyr), νCC (arom)
			**1558**	1541	1544				νCC (arom), νCC (pyr)
**1550**	1554	1548							νCC (arom), νCC (pyr)
						**1530**	(sh)	1540 (sh)	νCC (arom), νCC (pyr), νC=O (pyr)
**1532**	1518	-							scNH$_2^+$
**1517**	1494	1494							scCH$_2$ (piperazinium), scNH$_2^+$
**1500**	1494	1494							δCH (arom), scCH$_2$ (piperazinium)
						**1497**	1500	1492	δipCH (arom), scCH$_2$ (piper), νN2-C7
**1489**	1468	1467							δipOH, νCC (arom), νCC (pyr), δipCH, scCH$_2$ (piperazinium)
			**1487**	1498	1497				δip CH (arom)
**1479**	-	1455							scCH$_2$ (piperazinium), scNH$_2^+$
						**1476**	1480		scCH$_2$ (piper)
			**1476** **1470** **1460**	1485-1440	1485 - 1440				δCH$_2$ (piperazinium)
						**1465**	1454	1455	scCH$_2$ (piper), δN3H
**1457**	1447								δipOH, wNH$_2^+$, wCH$_2$ piperazinium
						**1441**		1440	scCH$_2$ (cyclo)
**1442**	1438 (sh)	1442							scCH$_2$ (piperazinium), scCH$_2$ (cyclo), δNH$_2^+$
			**1410** **1407**	(sh)	1413				δCH$_2$ (piperazinium), δNH$_2^+$
						**1395**			wCH$_2$ (piper)
**1391**	1399	1397							CH (pyr, cyclo)
			**1388** **1385**	1375	1382				δCH$_2$ (piperazinium), δNH$_2^+$
						**1387**	1399		wCH$_2$ (piper), δipCH (pyr), δCH (cyclo)
						**1381**		1384	wCH$_2$ (piper)
**1380**	1383	1384							δCH$_2$ (cyclo), tCH$_2$ (piperazinium), δipCH (pyr, arom)
						**1366**	1375		δCH (cyclo), δCH$_2$ (cyclo)

			**1371**	1364	1364				δCH (cyclo), δCH$_2$ (cyclo)
						**1362**	1358	1361	δCH (cyclo), δCH$_2$ (cyclo), δCH (pyr)
**1352**	1342	1343							δCH$_2$ (piperazinium)
**1343**									tCH$_2$ (piperazinium), δipCH (pyr), δipCH (arom)
						**1337**	1328		tCH$_2$ (piper), δNH, δipCH (arom)
			**1332**	1329					δCH$_2$ (piperazinium), δCH (arom, pyr, cyclo)
**1331**		1316							δipOH, νC2N1, δipCH (pyr), δipCH (arom), tCH$_2$ (piperazinium)
						**1320**	1311	1310	δipCH (arom), νC-COO$^-$
			**1312**	1310	1312				δCH$_2$ (piperazinium)
						**1309**	-	1300	δipCH (pyr), δCH (cyclo), δipCH (arom), ν$_s$COO$^-$
**1308**	1308	1299				**-**	-	-	δCH (cyclo), δipCH (pyr)
						**-**			δCH of all rings
						**1297**	1293		tCH$_2$ (piper), δipCH (pyr), ν$_s$COO$^-$
			**1292**	1284	-				ν$_s$COO$^-$, δipCH (pyr), δipCH (arom)
**1277**	1270	1272			-				tCH$_2$ (cyclo), δipCH (pyr, arom)
			**1269**	1260	-				νN1-C9, ν$_s$COO$^-$, δCH (pyr)
						**1252**	1256	1256	tCH$_2$ (piper), δipCH (arom), δipCH (pyr)
			**1248**	-	1264				δCH$_2$ (piperazinium), δipCH (arom)
**1231**	1220	1224							δipCH (arom, pyr), δCH (cyclo), δCH$_2$ (cyclo, piperazinium)
						**1215**	1217		δipCH (arom), δipCH (pyr), δCH$_2$ (piper, cyclo)

a) Wavenumbers calculated by DFT B3LYP 6-311G(d,p); b) ν = stretching, ν$_s$ = symmetrical stretching, ν$_{as}$ = antisymmetrical stretching, sc = scissoring, w = wagging, δ = bending, δip = bending in plane, δip = bending out of plane, t = twisting, sh = shoulder, arom = aromatic, cyclo = cyclopropyl, piper = piperazine, pyr = pyridone.

SI References

(1) Fabbiani, F. P. A.; Dittrich, B.; Florence, A. J.; Gelbrich, T.; Hursthouse, M. B.; Kuhs, W. F.; Shankland, N.; Sowa, H. Crystal Structures with a Challenge: High-Pressure Crystallisation of Ciprofloxacin Sodium Salts and Their Recovery to Ambient Pressure. *CrystEngComm* **2009**, *11* (7), 1396. https://doi.org/10.1039/b822987b.

(2) Turel, I.; Golobic, A. Crystal Structure of Ciprofloxacin Hydrochloride 1.34-Hydrate. *Anal Sci* **2003**, *19* (2), 329–330. https://doi.org/10.2116/analsci.19.329.
